# Supplementary material for: Proximal Pulmonary Artery Stiffening as a Biomarker of Cardiopulmonary Aging
Source: Aging Cell. 2026 Jan 27;25(2):e70383. doi: 10.1111/acel.70383 (PMC12836046; doi:10.1111/acel.70383)
Supplement: Supplementary file 1 — Data S1: acel70383‐sup‐0001‐supinfo.docx. [file ACEL-25-e70383-s001.docx]

**Supplemental Information**

**Proximal Pulmonary Artery Stiffening as a Biomarker of Cardiopulmonary Aging**

Ruben De Man^1,*^, Zhongyu Cai ^1,2,*^ , Pramath Doddaballapur^3^ , Nicole Guerrera^4^ , Alexandria Regan^1^ , Liqin Lin^1,5^ , Erica Schwarz^6^ , Aurelien Justet^1,7^ , Nebal S. Abu Hussein^1^ , Jack Di Palo^1^ , Cristina Cavinato^8^ , Micha Sam B. Raredon^9,10^ , Paul M. Heerdt^9^ , Inderjit Singh^1^ ,

Xiting Yan^1,2^ , Min-Jong Kang^1^ , Danielle R. Bruns^11^ , Patty J. Lee^12^ , George Tellides^10,13,14^ , Jay D. Humphrey^5,10^ , Naftali Kaminski^1^ , Abhay B. Ramachandra^15,+^,Edward P. Manning^1,14,+^

*,+ authors contributed equally

Corresponding author email address: edward.manning@yale.edu


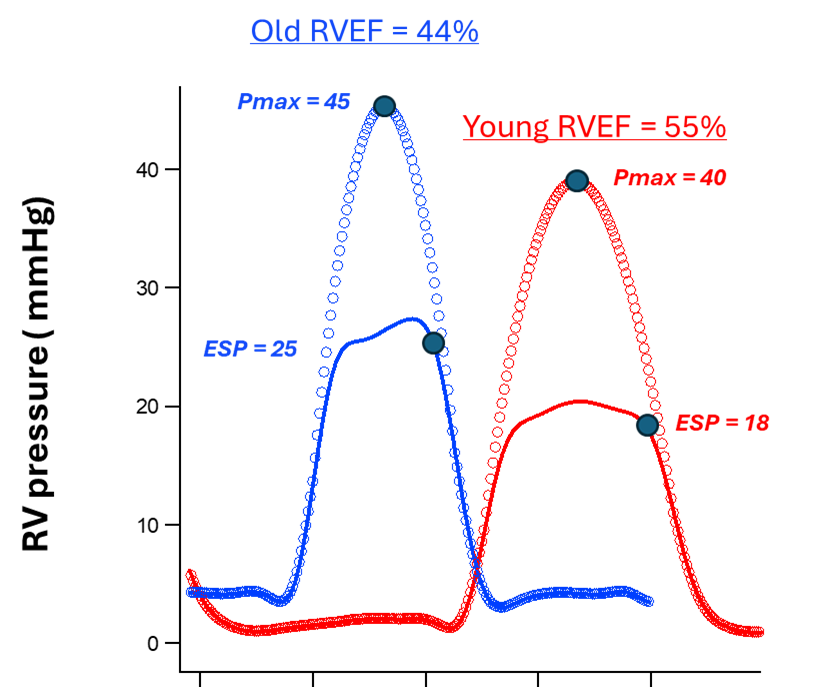


**Supplemental Figure S1:** Right ventricle (RV) mechanical characteristics. Standardized sampling and filters to create an example comparison of young (n=3) and old (n=3) RV pressure waveforms from which ejection fraction was calculated using our pressure-based method^1^, where EF is calculated as (Pmax-ESP)/Pmax, or 1-(ESP/Pmax). Peak and end-systolic pressures are elevated in older mice. Pmax = maximum pressure; ESP = end-systolic pressure.

**Remark 1**: Histological measurements of RV free wall thickness revealed significant increase in older hearts (young = 543.7 +/- 30.65 um versus old = 646.2 +/- 27.15 um, p = 0.022), similar to the trend of increased RV free wall thickness from echocardiographic measurements in Supplemental Table S1 (young = 0.31 +/- 0.03 mm versus old = 0.38 +/- 0.03, p 0.15; note that measurements from young male mice were not obtained due to poor visualization). Histological RV measurements of RV area found a significant increase in the cross-sectional area of the RV of old mice (young = 3.52 +/- 0.15 mm^2^ versus old = 7.11 +/- 0.84, p < 0.001) similar to significance enlargement of the RV area in systole (p = 0.0003) and diastole (p = 0.0007) from echocardiographic measurements in Supplemental Table S1.

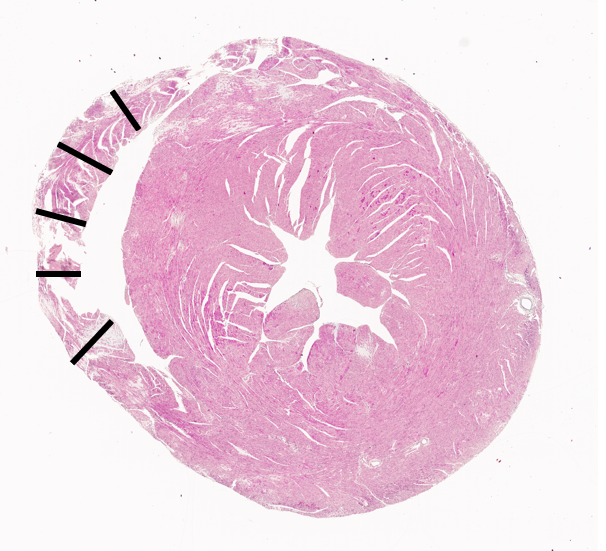

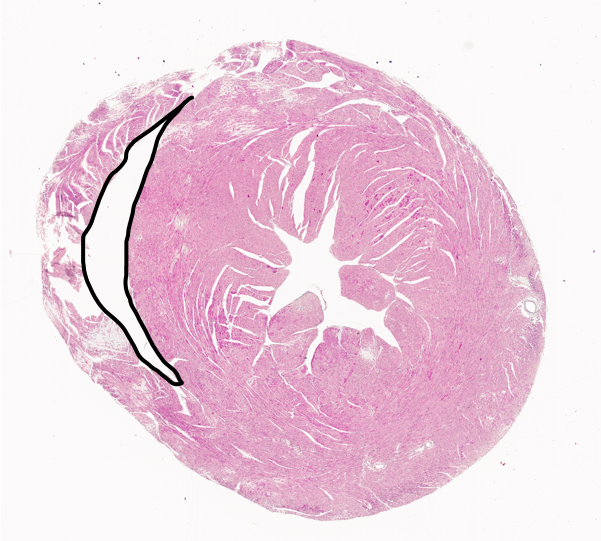


RV Free Wall Thickness RV Area

**Remark 2**: We found that this increase in RV thickness of hearts from old mice associates with a significant increase in the proportion of collagen (young = 9.58 +/- 0.3% versus old = 10.28 +/- 0.3%, p = 0.04) and a trend toward increased cytoplasm (young = 81.25 +/- 1.3% versus old = 83.12 +/- 1.2%). These values reflect an abundance of cellular cytoplasm of cardiomyocytes with a relatively small amount of collagen and trace amounts of elastin, which agrees with the range of data in the literature^2-5^. Collagen and elastin make up a small percentage of the heart by mass, and our data is comparable to data in the literature^3, 5^**.**

**Supplemental Figure S2:** Additional associations of changes in right ventricular and lung mechanics and changes in circumferential stiffness of the proximal pulmonary artery.

**Remark 3**: We fixed lungs from ten young mice (five 3-month-old males and five 3-month-old females) and ten old mice (three 24-month-old males, two 28-month-old males, four 22-month-old females, and one 28-month-old female) at 20 cmH2O airway pressure and analyzed the characteristics of the lung parenchyma and distal vasculature. Although the blood vessels were unloaded, comparative measurements under similar conditions have proven beneficial in models of lung disease^6^. We found increased alveolar size in lungs of old mice, demonstrating a trend toward age-related rarefaction, similar to age-related changes described by others^7^:

Solid = female, Hollow = male, Gold = young, Purple = old


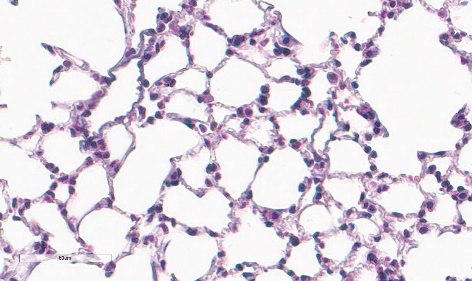

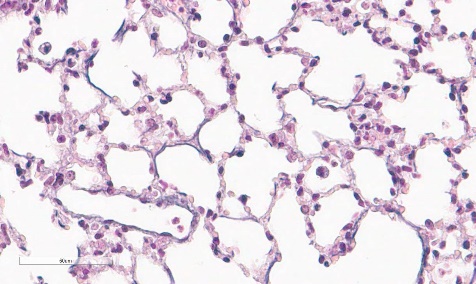


( 60um) 3 month Male (40x Movat) 24 month Male

**Remark 4**: We hypothesize that similar phenomenon may be occurring in the smooth muscle cells of distal pulmonary arteries as what we observe in the proximal pulmonary arteries. Recall Figure 2, Figure 3, and Supplemental Figure S5 that there is a trend toward fewer number of cells in the media of older proximal pulmonary arteries despite an increase in medial thickening and similar ability to contract. This suggests that there may be age-related changes in the phenotype of smooth muscles cells to maintain contractility in response to age-related changes in arterial wall mechanics. Our single cell RNA sequencing results support the concept of age-related changes of phenotype of SMC’s. We suspect that a similar age-related phenomenon may be occurring in SMC’s of the distal pulmonary arteries, however, we have not tested this hypothesis yet, as these are outside the scope of the current study.

We performed compositional analysis of lung parenchymal and found that lung parenchyma from older mice had significantly more fibrosis (proportion of collagen in young lungs = 38.1 +/- 1.0 % versus old lungs = 48.2 +/- 0.7 %, p = 0.0015). There is a significant decrease in the proportion of elastin in old compared to young lungs (young = 10.9 +/- 0.5% versus 4.2 +/- 0.3 %, p < 0.0001). The proportions of collagen and elastin are comparable to values reported in the literature for both animal models and humans^8-14^. However, we emphasize the age-related changes associated with the mechanical and hemodynamic changes observed in the pulmonary circulation.

**Remark 5**: Distal pulmonary arteries in lungs of old mice significantly increase in diameter:

Solid = female, Hollow = male, Gold = young, Purple = old


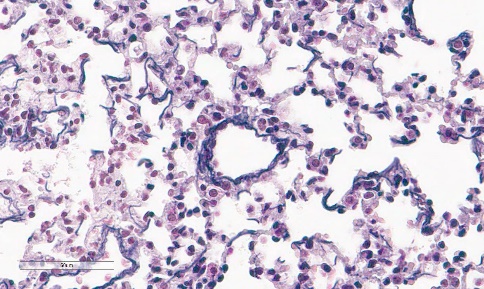

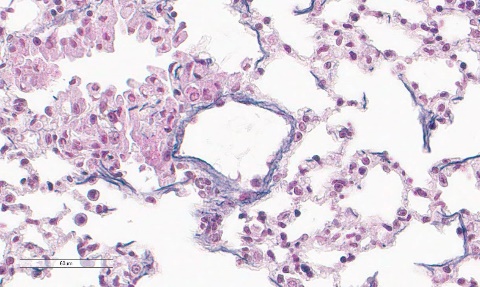


( 60um) 3 month Male (40x Movat) 24 month Male

**Remark 6**: We believe this is due to the damaging effects on endothelial cells in capillaries resulting from increased PWV. Consequently, there is alveolar epithelial damage and rarefaction of alveoli. This functionally results in impaired diffusion capacity, as shown in Supplemental Figure S2. We also believe that increased PWV affects endothelial cells in the distal circulation, which may explain the significant increase in intimal thickening observed in distal pulmonary arteries in older mice, as shown below.

Solid = female, Hollow = male, Gold = young, Purple = old


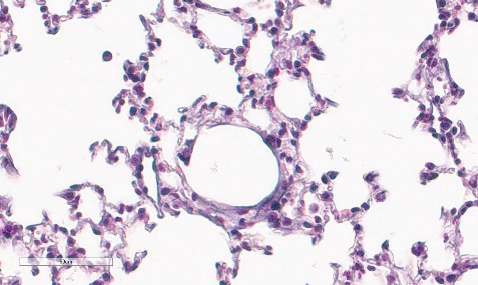

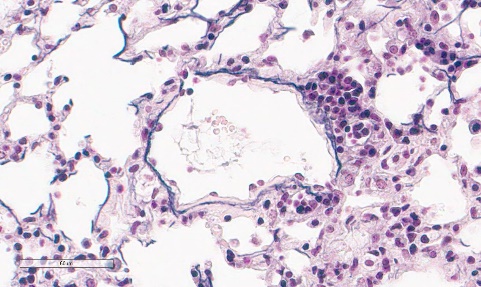


( 60um) 3 month Male (40x Movat) 24 month Male

Additional examples of intimal thickening:


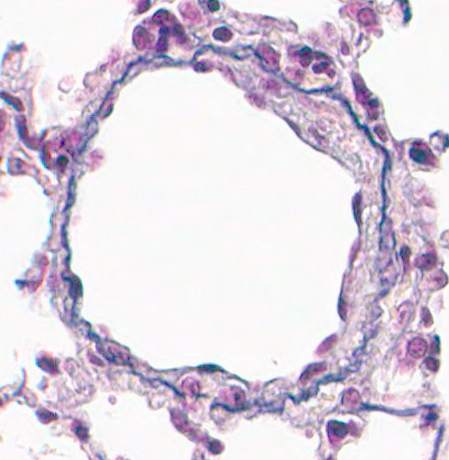

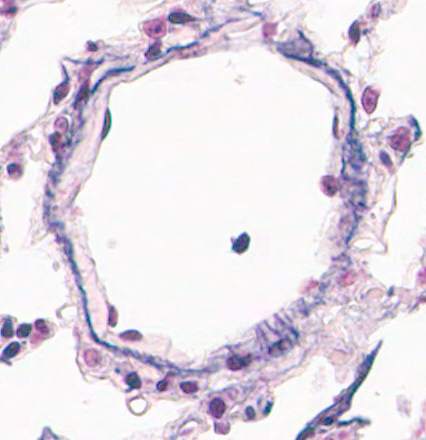

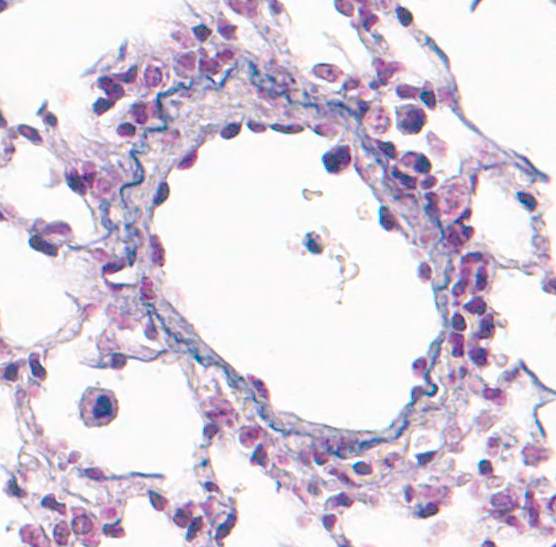


28-month Female 24-month Male 24-month Male

**Remark 7**: There are signs of increased muscularization of the distal pulmonary arteries:

Solid = female, Hollow = male, Gold = young, Purple = old


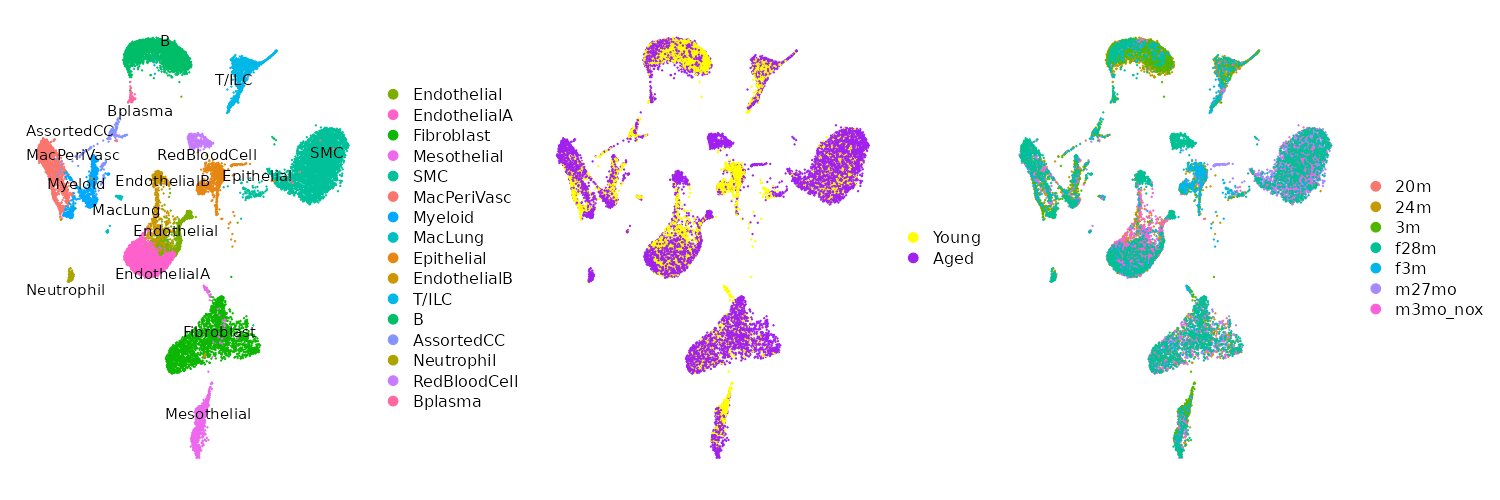


**Supplemental Figure S3:** Uniform manifold approximation and projection (UMAP) embedded as cell type, age grouping, and individual samples.

**Remark 8**: A heatmap demonstrates gene markers used to define cell types.


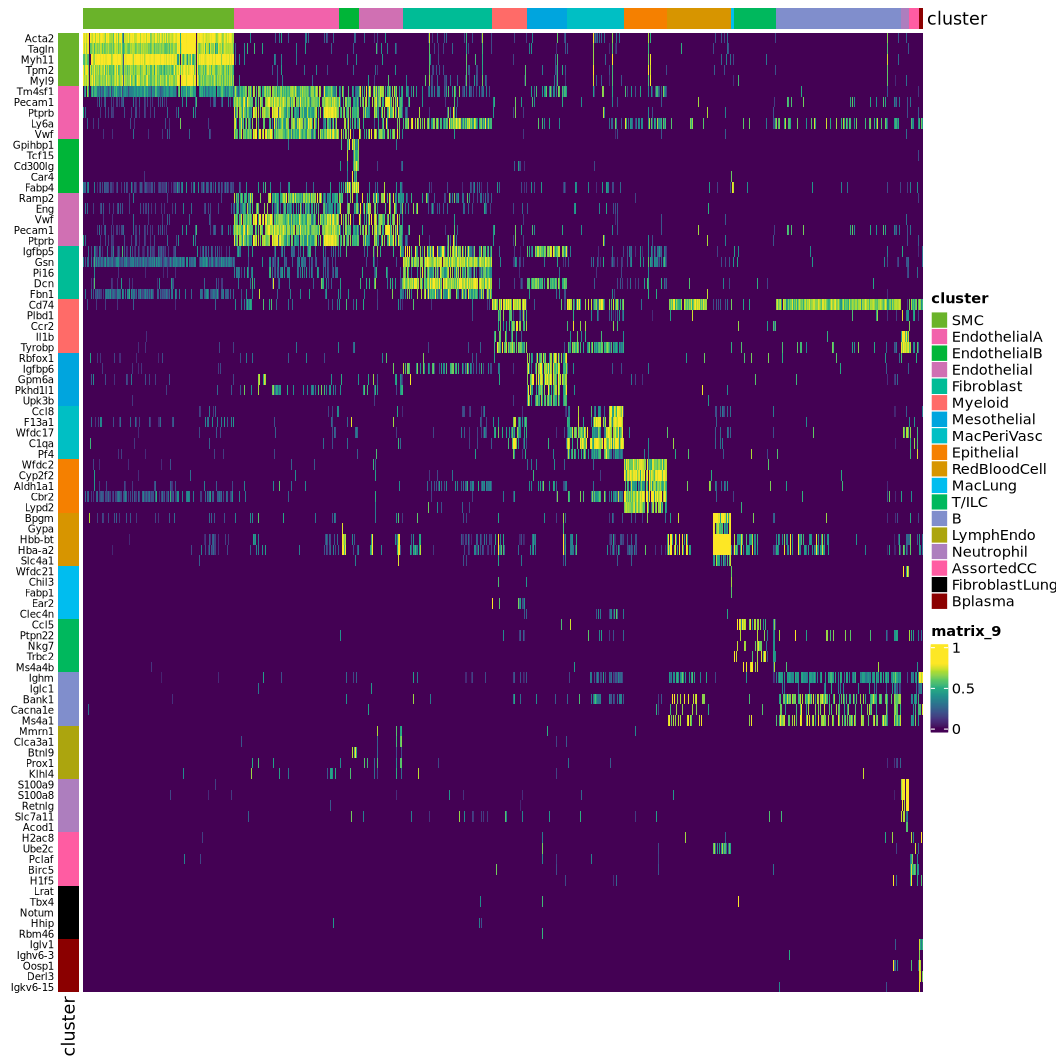


Left: Expression of differentiation markers in cell types. Right: Cell proportions are similar between young and old age groups.


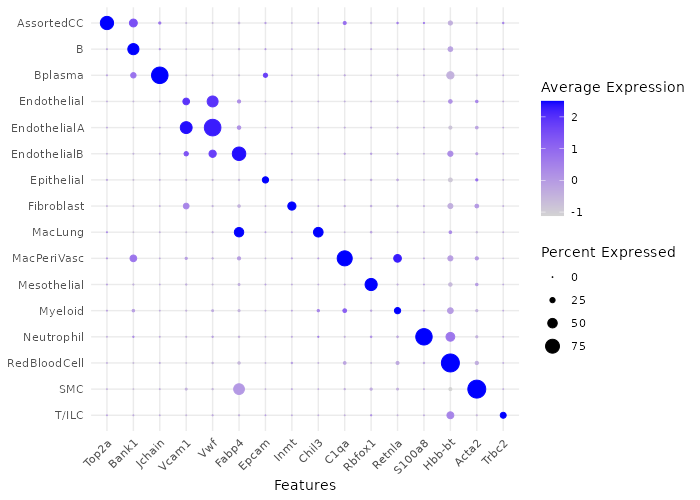

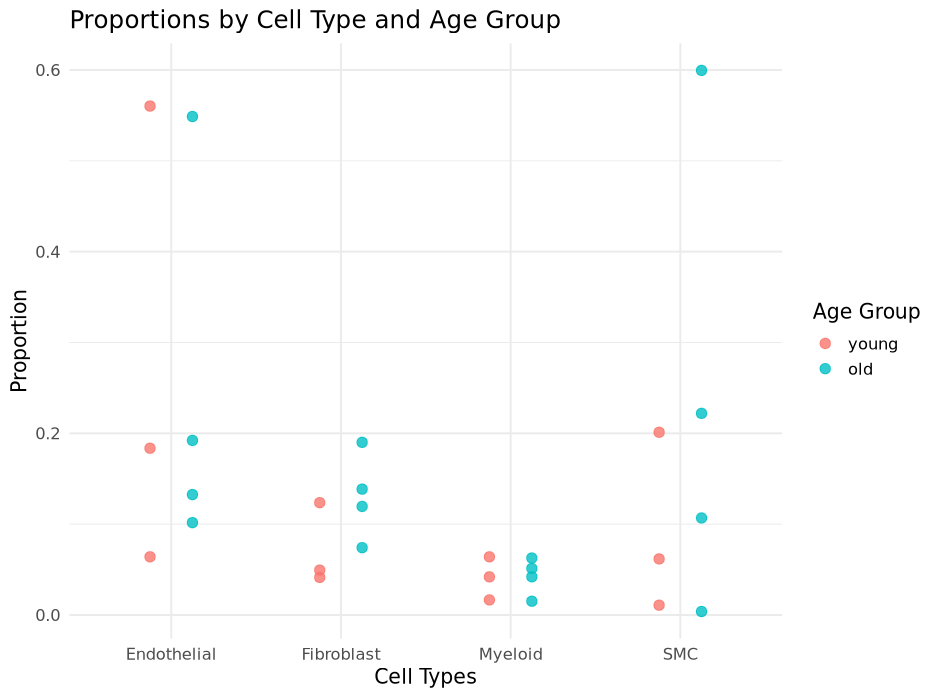


Differentiating general monocytic cells (Myeloid), macrophages that reside in vascular wall (MacPeriVasc), and macrophages that tend to reside in alveoli (MacLung).


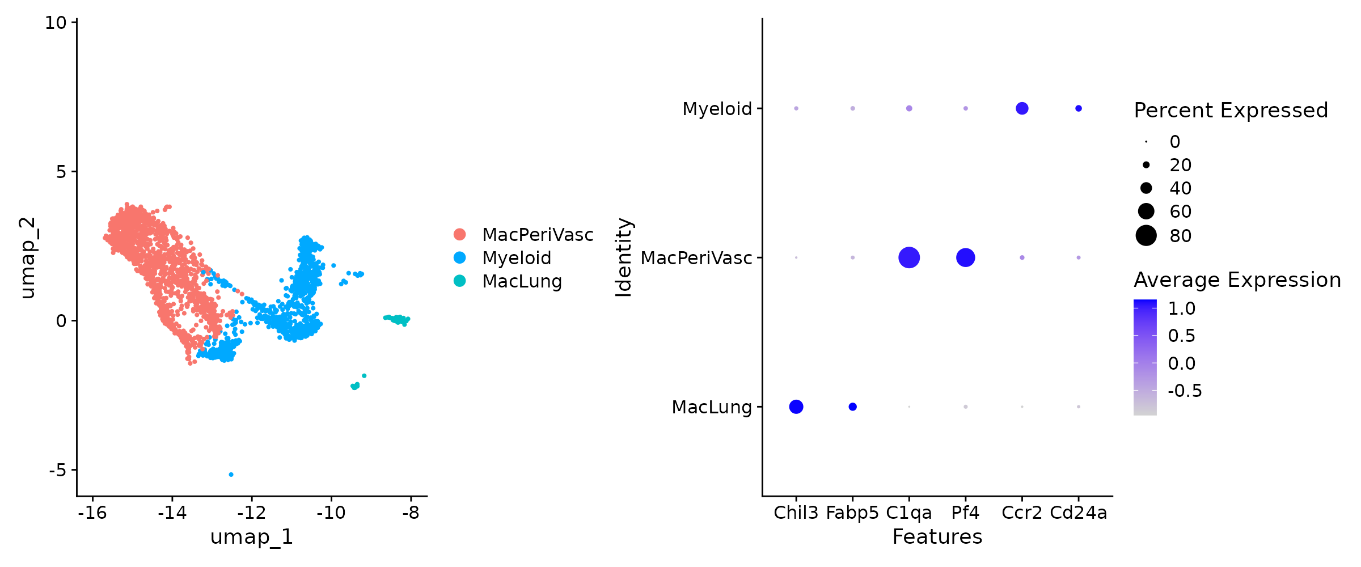


SenMayo senescence score distribution and thresholding.


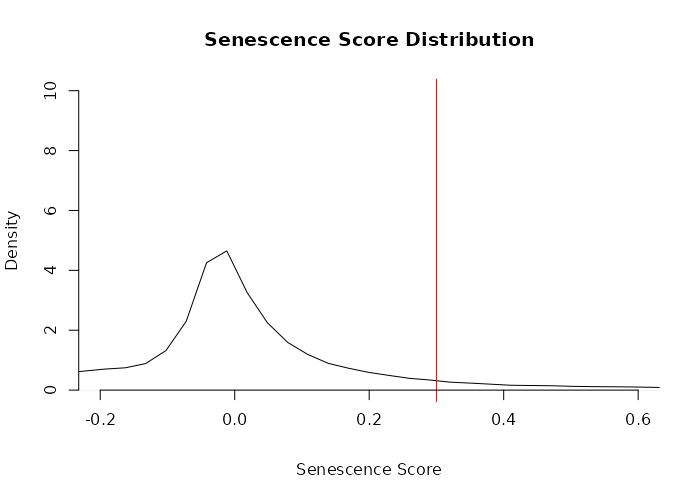


Left: Plotting of High SenMayo expressing cells on UMAP. Right: UMAP plot of NICHES cell signaling data.

**
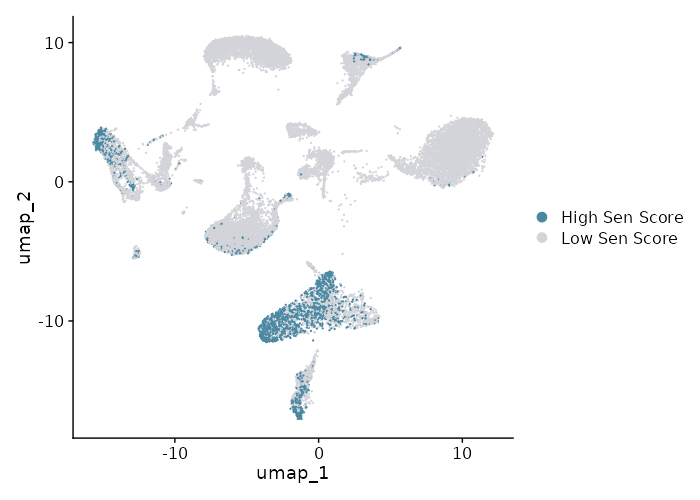

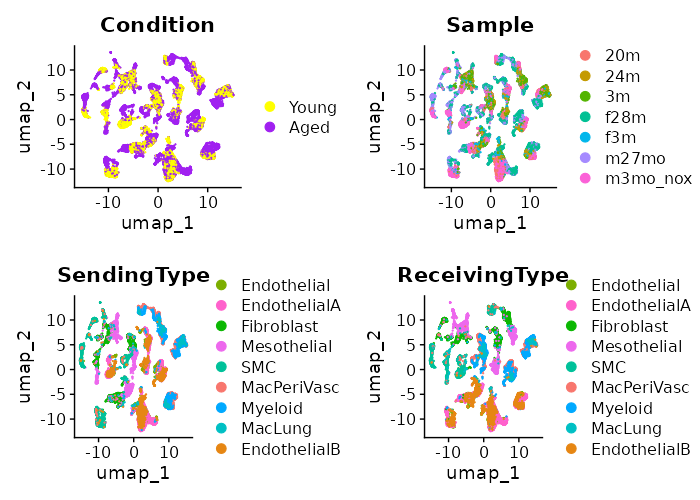
**

**Remark 9**: The gene expression profile of SenMayo markers are grouped into functional categories and signaling pathways. Cell-specific categories that differed between young and old mice include:

- Perivascular macrophages: Intercellular signaling, growth factors, and matrix metalloproteases
- Fibroblasts: Cytokines/chemokines, protein modifying enzymes, growth factors and intercellular signaling
- Smooth Muscle Cells: matrix metalloproteases and transmembrane signaling receptors


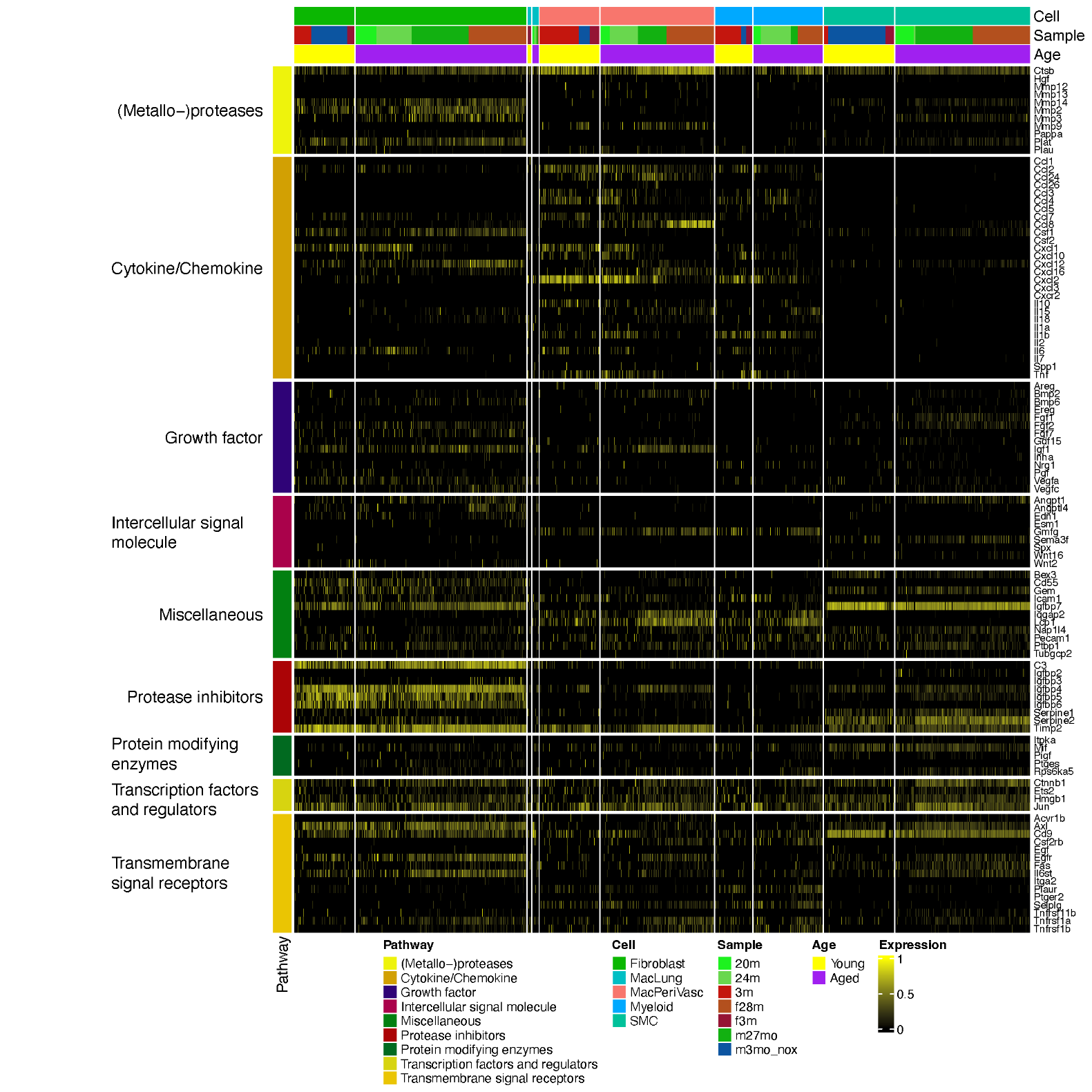


We found a significant increase in the proportion of p21+ cells in hearts (p = 0.008), proximal pulmonary arteries (p < 0.001), and lungs (p = 0.046) from old mice compared to young. We found similar increases in markers of senescence reported in cardiac aging^15, 16^ as well as lung aging and injury^17-19^. However, we are not aware of previously reported reports of senescence of the proximal pulmonary artery in mice or humans. The percentage of p21+ cells in young and old proximal pulmonary arteries of mice appears greater than the heart and lung tissues.


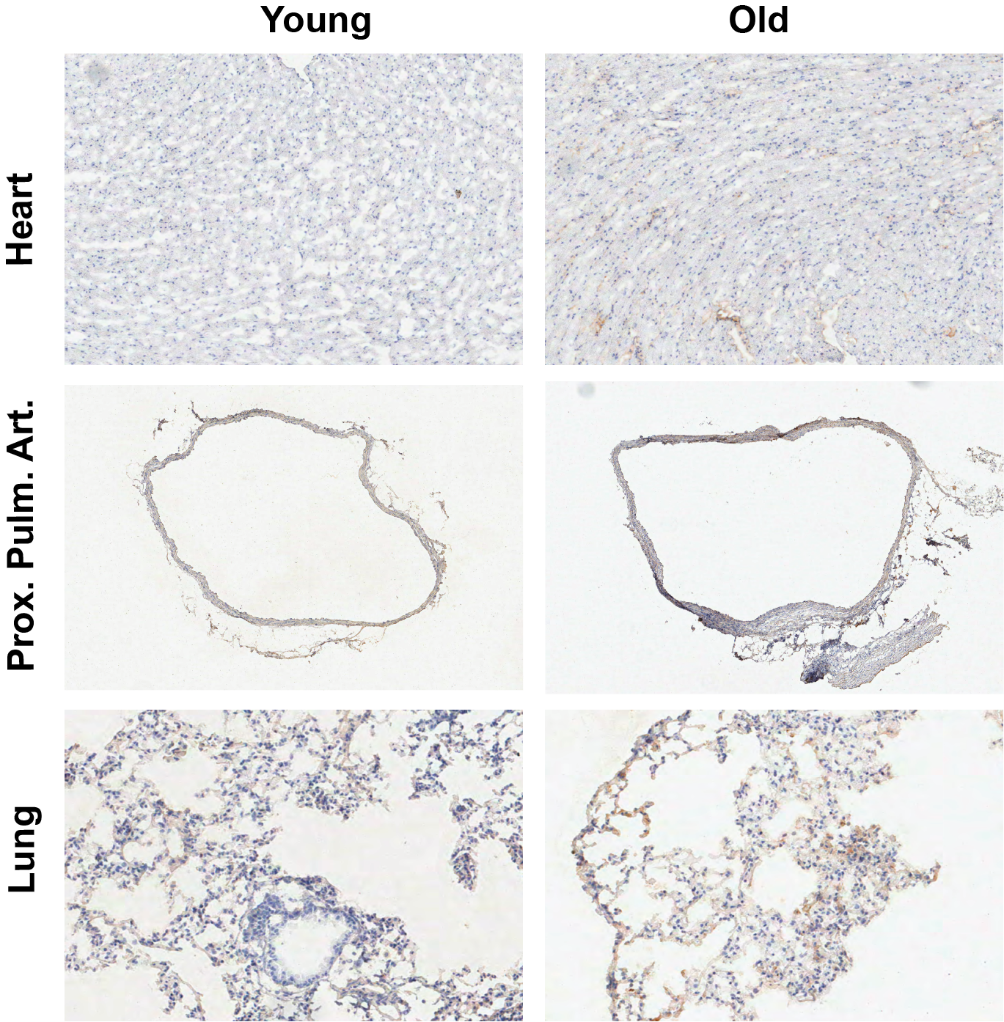

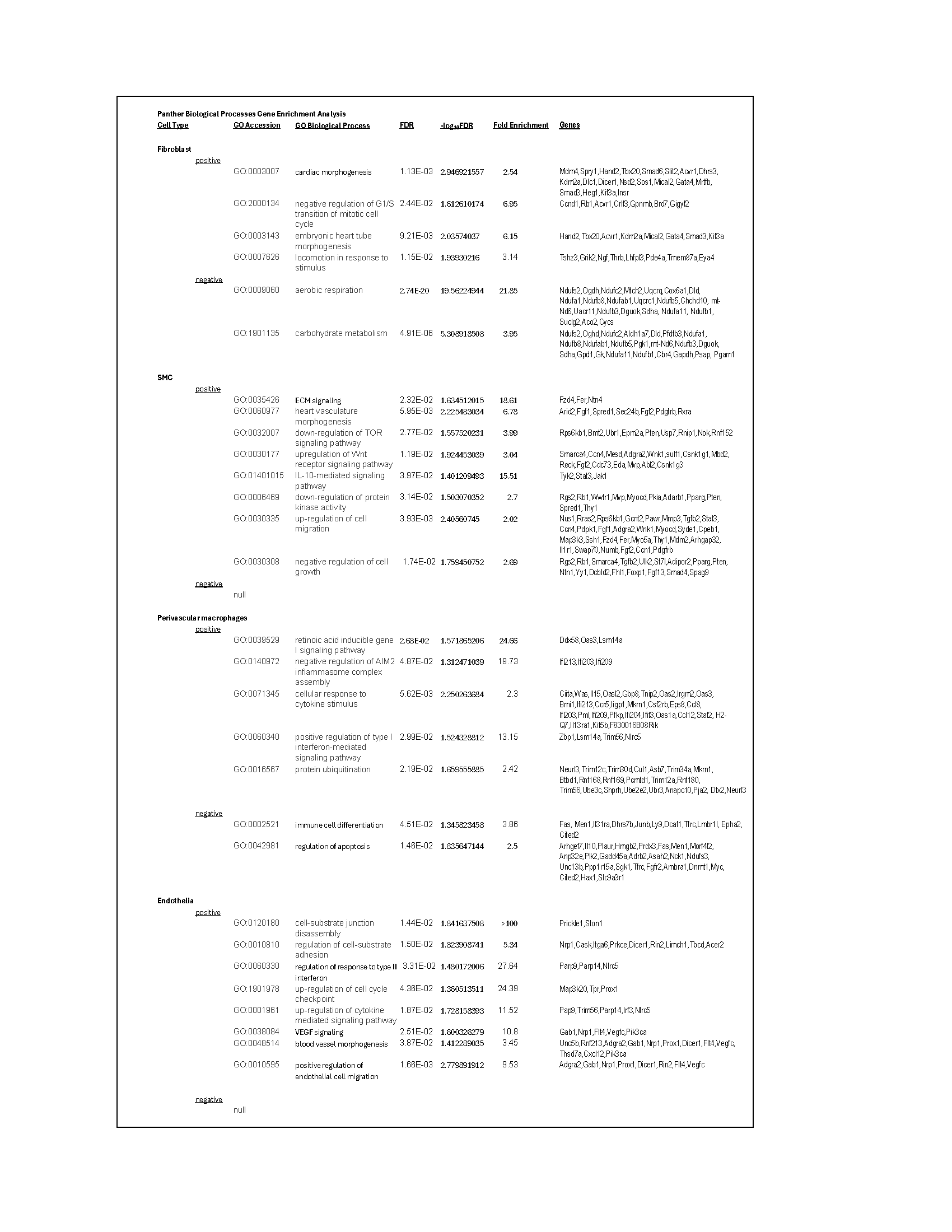


**Supplemental Figure S4.** Gene Enrichment Analysis – Biological Processes (Panther)


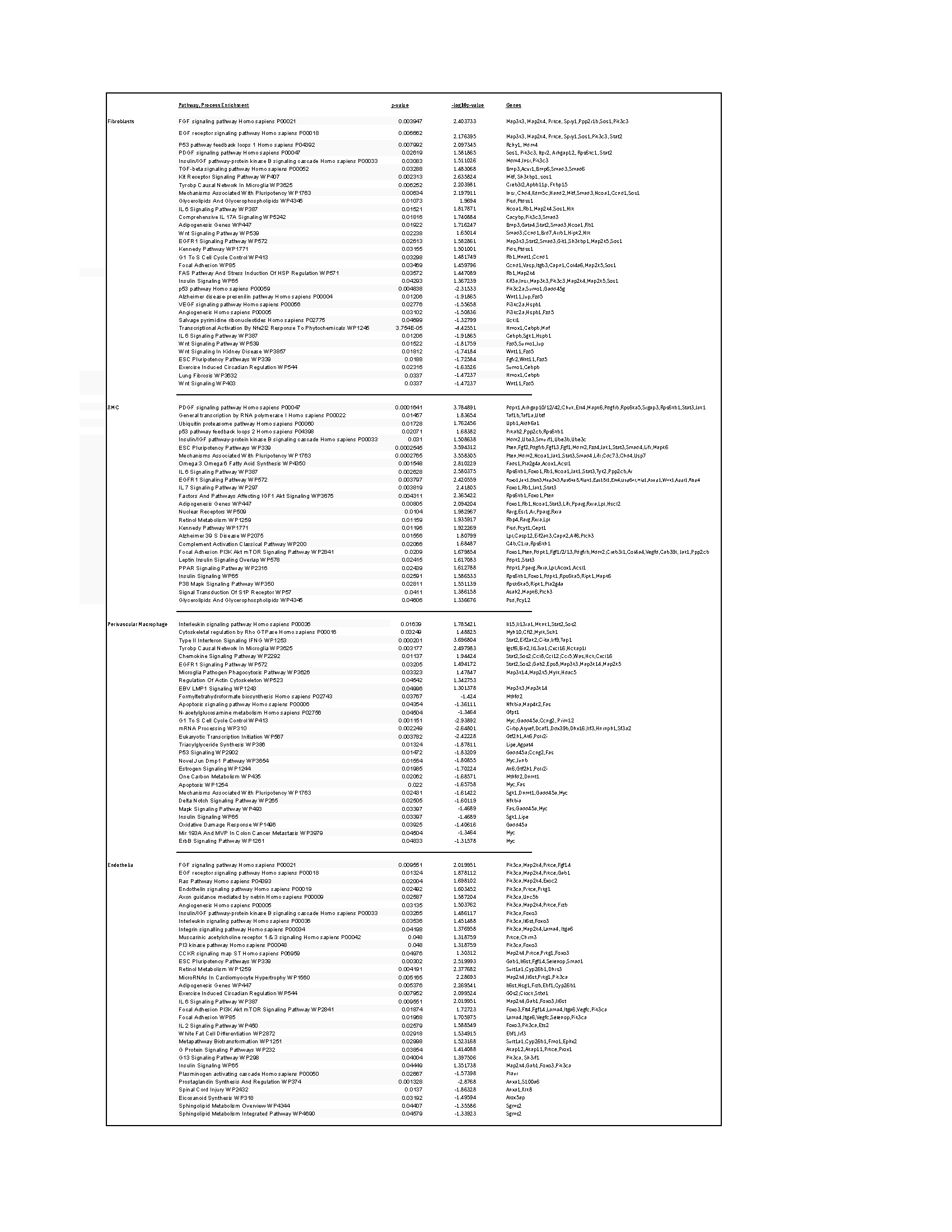


**Supplemental Figure S4-2: Gene Enrichment Analysis** – Pathways (Panther & WikiPathways Mouse)

Panther 2016:


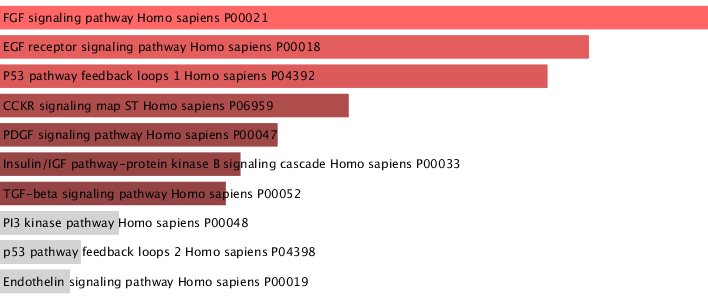

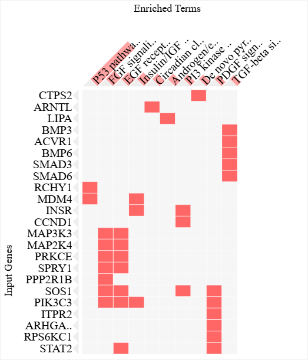


WikiPathways 2024 Mouse:


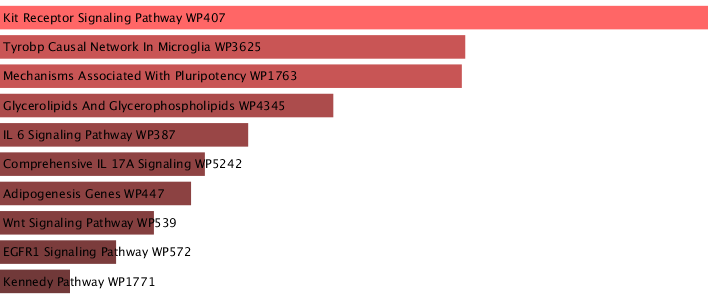

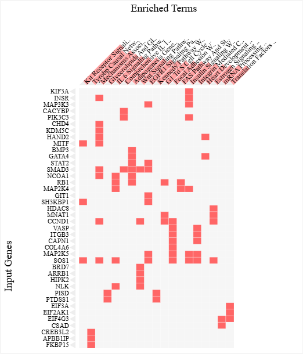


**Supplemental Figure S4-3:** Fibroblast positive gene enrichment analysis using Enrichr

Panther 2016:


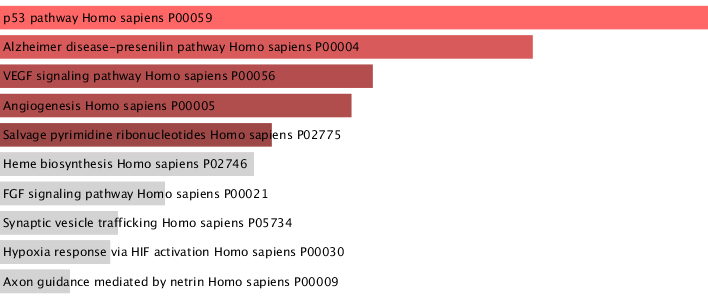

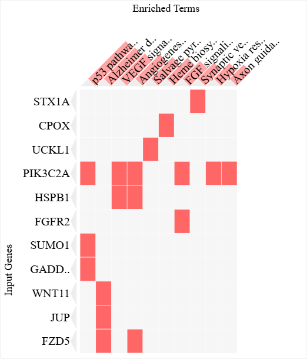


WikiPathways 2024 Mouse


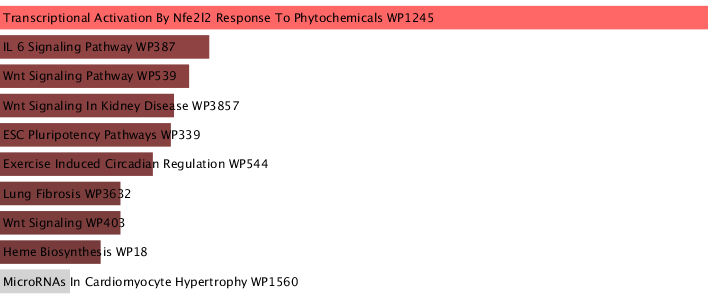

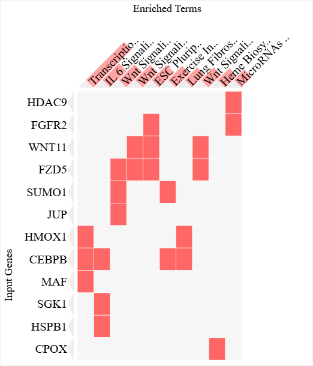


**Supplemental Figure S4-4:** Fibroblast negative gene enrichment analysis using Enrichr

Panther 2016:


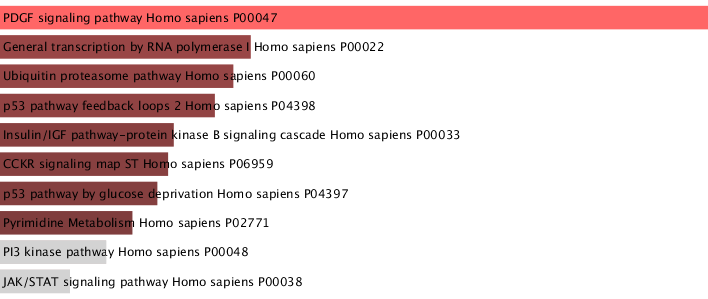

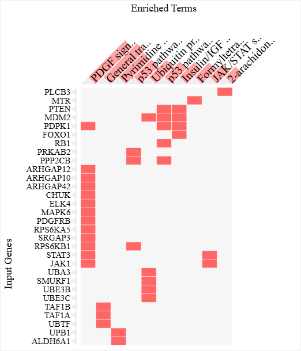


WikiPathways Mouse 2024


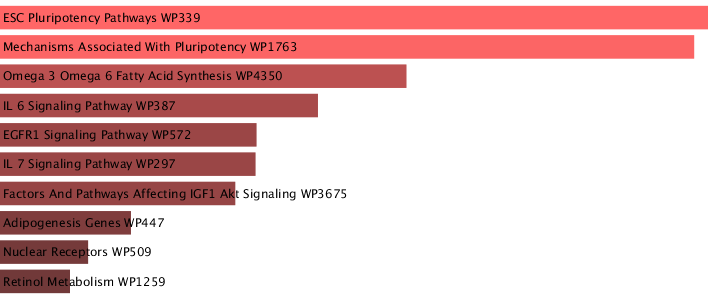

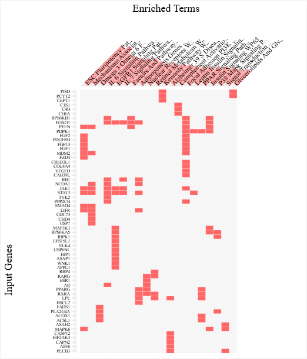


**Supplemental Figure S4-5:** SMC positive gene enrichment analysis using Enrichr

Panther 2016:


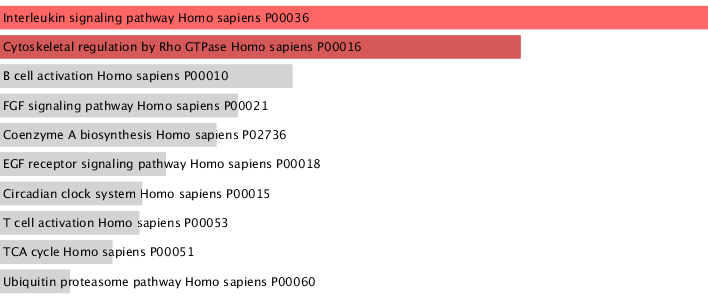

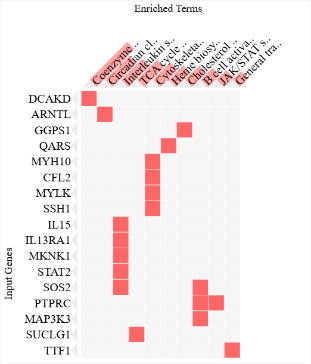


Mouse WikiPathway 2024:


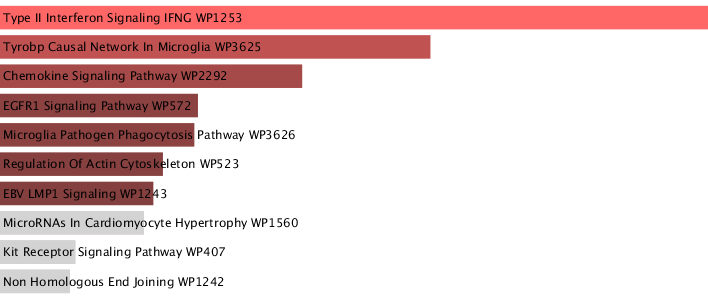

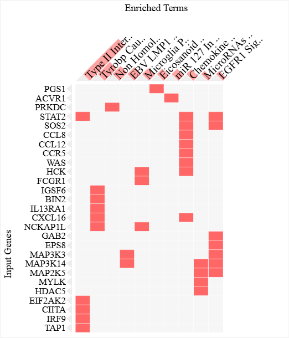


**Supplemental Figure S4-6:** Perivascular Macrophage positive gene enrichment analysis using Enrichr

Panther 2016:


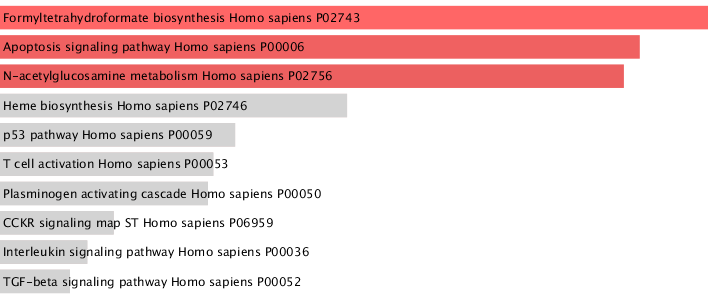

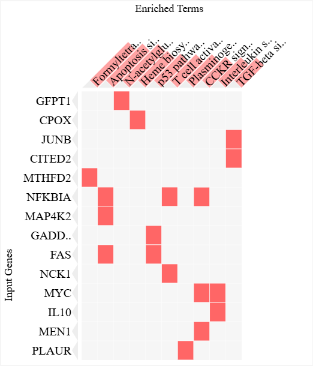


WikiPathways Mouse 2024:


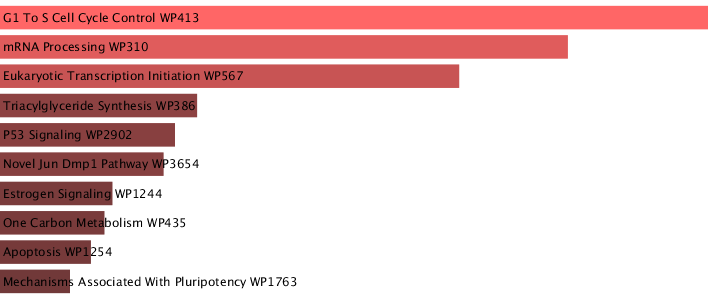

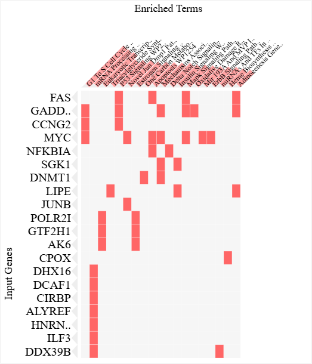


**Supplemental Figure S4-7:** Perivascular Macrophage negative gene enrichment analysis using Enrichr

Panther 2016:


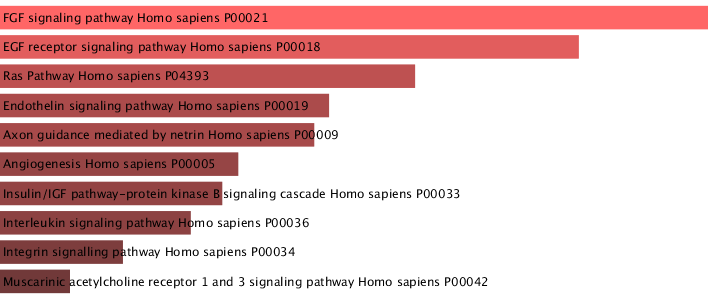

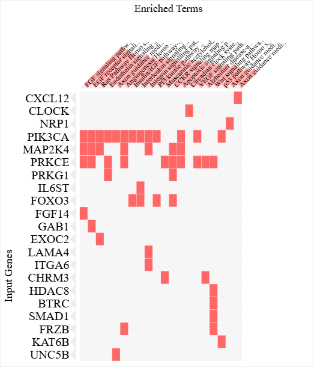


WikiPathways 2024 Mouse:


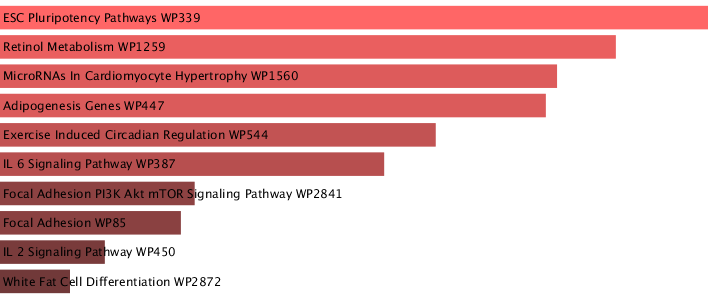

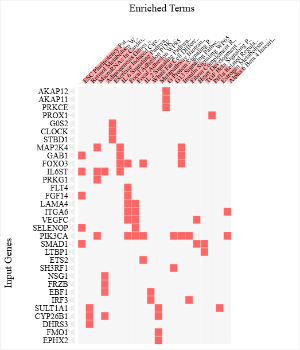


**Supplemental Figure S4-8:** EC positive gene enrichment analysis using Enrichr

Panther 2016:


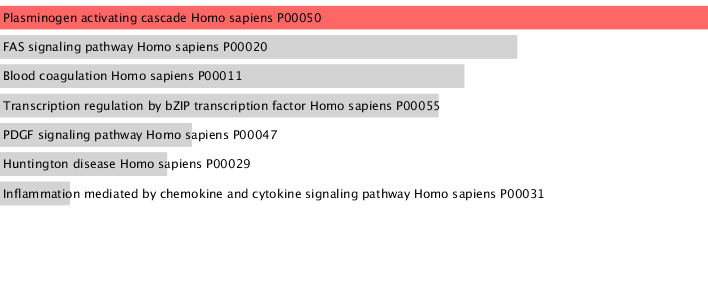

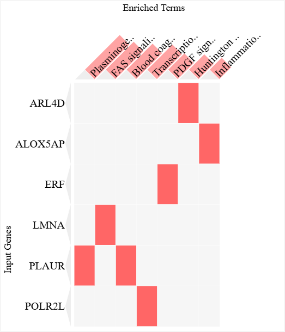


WikiPathways 2024 Mouse:


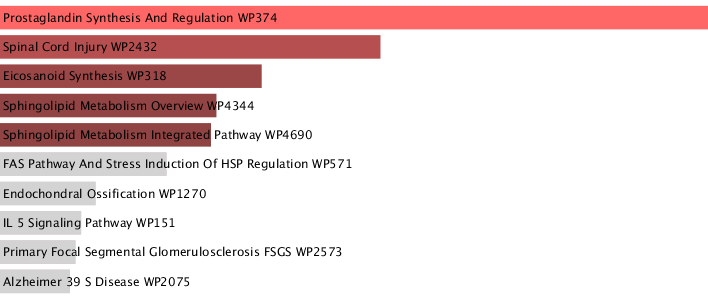

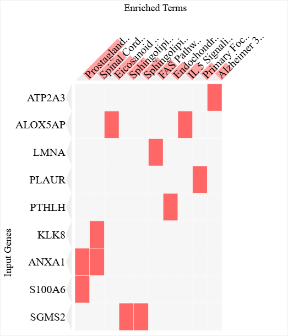


**Supplemental Figure S4-9:** EC negative gene enrichment analysis using Enrichr

**
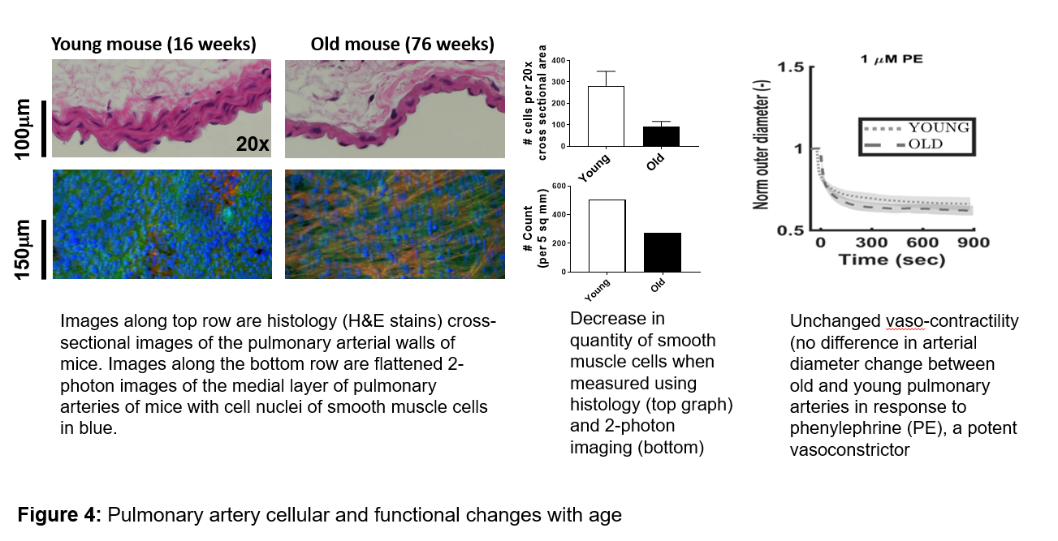
**

**Supplemental Figure S5.** Left: H&E stains of proximal pulmonary arteries from mice. Images on the bottom row are representative z-slices of the medial layer of flattened 2-photon images of proximal pulmonary arteries from mice in which the cell nucleir of SMC’s are stained with syto 17. Right: There is no significant decrease in the density of smooth muscle cells of proximal PA’s from young and old mice when quantified by histology (H&E). This is consistent with our findings from 2-photon imaging (cf. Figure 2D).


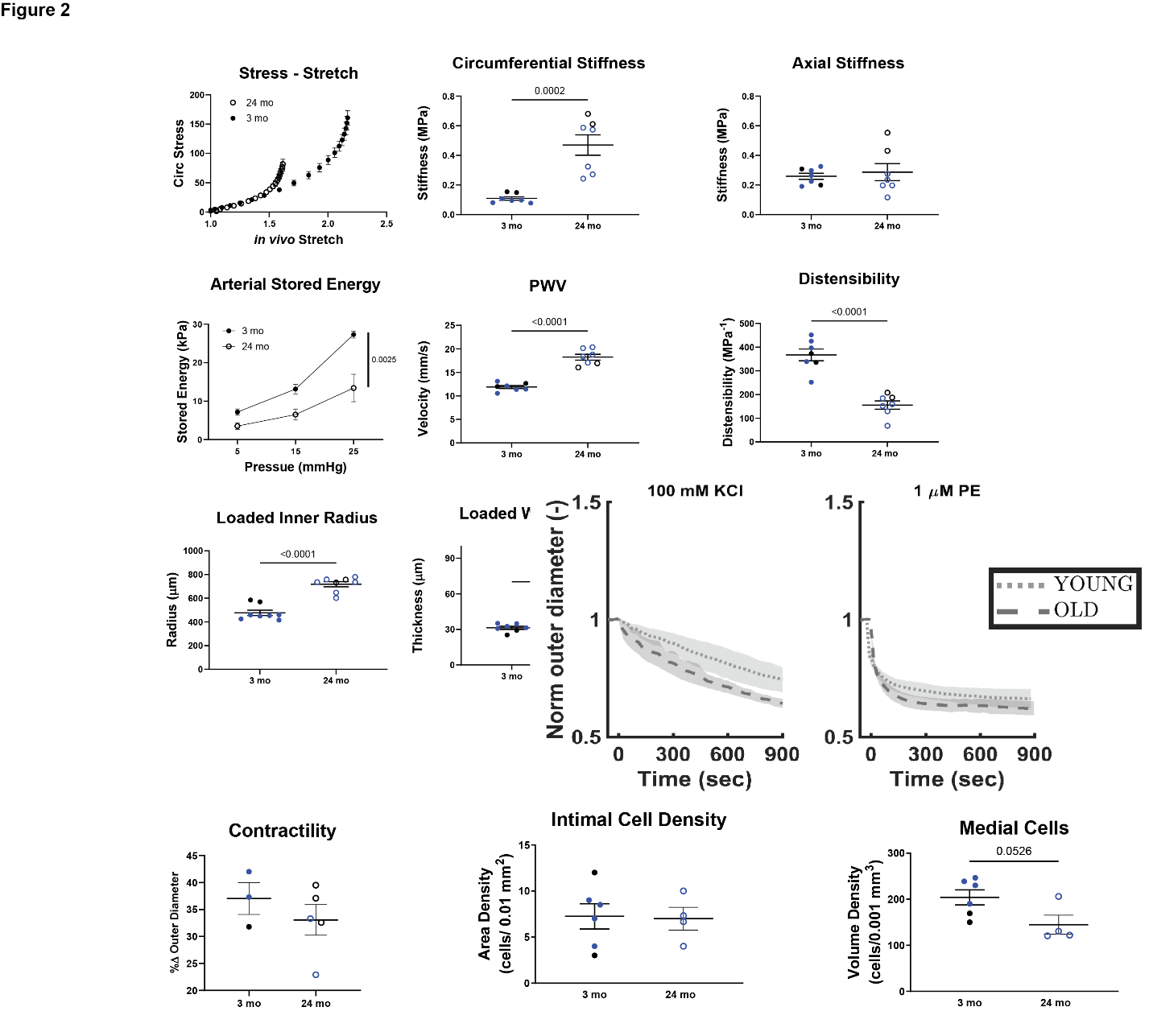


**Remark**: We found no significant difference in the vaso-contractility (based on change in of arterial diameter from baseline) in response to potassium chloride (KCl) or phenylephrine (PE).

**Supplemental Table S1:** Additional cardiac RV data from ultrasound measurements. RV-right ventricle, RA- right atrium, PAT – pulmonary acceleration time, PET- pulmonary ejection time, FAC – fractional area change, CO- cardiac output, SV- stroke volume, TAPSE - Tricuspid Annular Plane Systolic Excursion


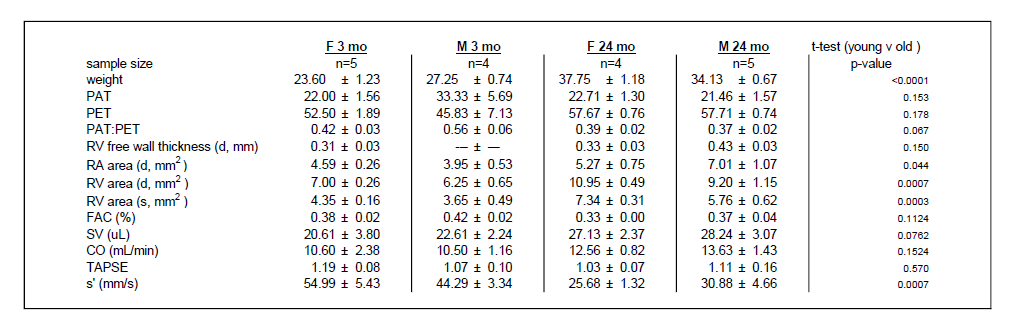


**Supplemental Table S2:** Additional lung mechanics.


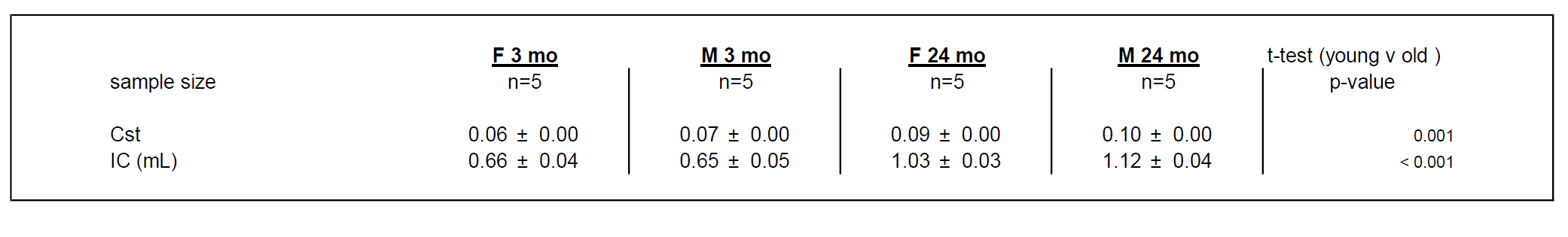


**Supplemental Table S3:** Biomechanical table and material properties.
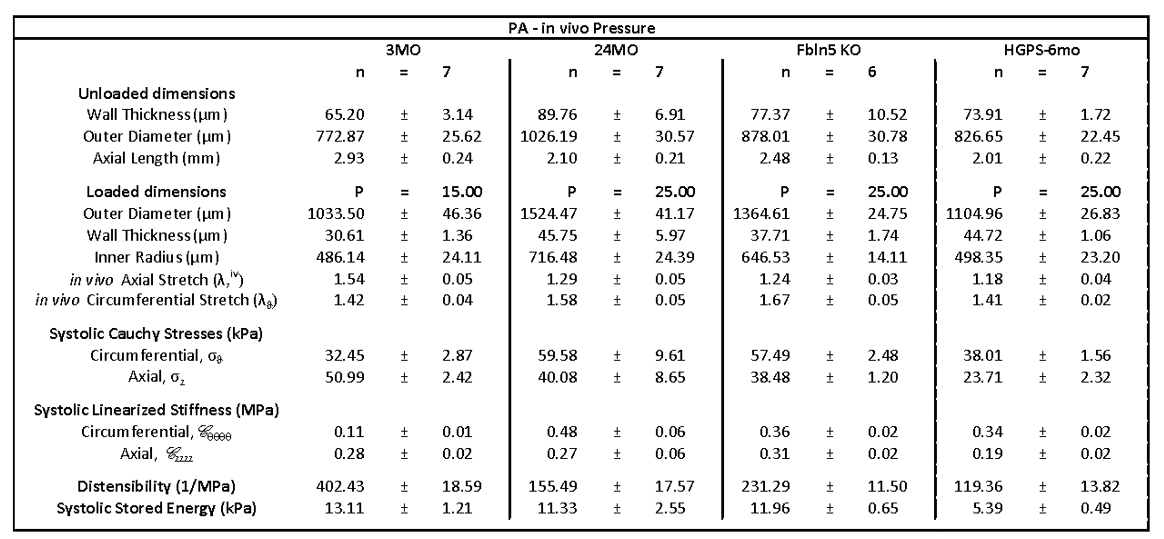


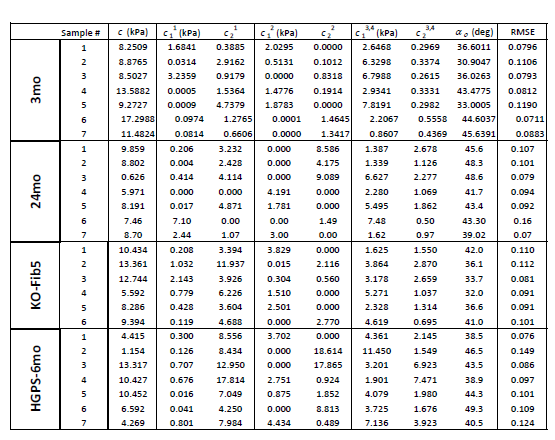


**Supplemental Table S4:** Top 50 genes differentially expressed genes from GLME model by cell type. Gene name is the first column. Β_0_ = intercept coefficient. Β_1_ = slope coefficient. Disp = dispersion parameter, Sigma = standard deviation, P.Beta = p-value, AIC = Akaike information criterion, fdrP = FDR-corrected p-value.

**Fibroblast: gene expression, positive correlation (β_1_) with age**

| **gene** | **Beta0** | **Beta1** | **Disp** | **Sigma** | **P.Beta** | **AIC** | **fdrP** |
| --- | --- | --- | --- | --- | --- | --- | --- |
| Itih5 | -9.2953 | 0.731 | 0.7305 | 7.02E-05 | 5.97E-08 | 4587.9208 | 3.52E-05 |
| Vps13d | -10.4628 | 1.1157 | 8.1092 | 2.70E-05 | 8.71E-08 | 2858.1634 | 4.97E-05 |
| C130026I21Rik | -10.1638 | 1.2109 | 1.2616 | 1.22E-01 | 1.21E-07 | 3724.8345 | 5.87E-05 |
| Prelp | -9.1327 | 0.7997 | 1.6873 | 1.00E-01 | 1.50E-07 | 5210.2008 | 7.07E-05 |
| Pknox2 | -11.4461 | 1.705 | 1.2443 | 6.29E-05 | 5.65E-07 | 2279.0031 | 2.22E-04 |
| Kdm2a | -9.7268 | 0.7347 | 4.9688 | 1.94E-02 | 1.22E-06 | 3565.9903 | 4.28E-04 |
| Mllt10 | -9.5271 | 0.7147 | 4.5736 | 6.15E-02 | 3.08E-06 | 3951.8103 | 8.48E-04 |
| Mdm4 | -10.8094 | 1.1451 | 4.4466 | 3.32E-05 | 3.89E-06 | 2400.4257 | 9.98E-04 |
| Trim8 | -10.384 | 0.93 | 4.7436 | 4.20E-05 | 4.07E-06 | 2736.8369 | 9.98E-04 |
| Ngf | -10.5408 | 1.0882 | 0.4094 | 6.06E-05 | 4.15E-06 | 2739.5772 | 9.98E-04 |
| Lipa | -10.5112 | 0.9936 | 2.5004 | 5.12E-05 | 4.16E-06 | 2659.9914 | 9.98E-04 |
| Klhl29 | -10.0326 | 0.8381 | 0.7139 | 3.16E-05 | 4.36E-06 | 3236.8469 | 1.03E-03 |
| Rab7b | -11.0042 | 1.2496 | 3.138 | 2.85E-05 | 4.78E-06 | 2159.1945 | 1.11E-03 |
| Gypc | -11.8501 | 1.8889 | 10.4464 | 2.00E-02 | 5.37E-06 | 1928.2471 | 1.23E-03 |
| Ilrun | -10.55 | 0.9862 | 663.4363 | 3.54E-14 | 5.53E-06 | 2526.967 | 1.25E-03 |
| Map2k5 | -11.095 | 1.3961 | 3.7014 | 1.09E-01 | 5.77E-06 | 2406.5062 | 1.29E-03 |
| Uba6 | -10.8086 | 1.1133 | 4.7893 | 3.33E-05 | 7.14E-06 | 2307.3531 | 1.55E-03 |
| Ncoa1 | -9.3136 | 0.6958 | 2.7014 | 8.82E-02 | 9.06E-06 | 4525.6458 | 1.85E-03 |
| Zfp950 | -10.7511 | 1.1781 | 2.259 | 6.62E-02 | 9.24E-06 | 2577.1546 | 1.86E-03 |
| Zscan26 | -11.3383 | 1.4025 | 14.191 | 1.55E-03 | 1.20E-05 | 2014.4047 | 2.26E-03 |
| Bmp6 | -10.765 | 1.1766 | 0.2241 | 5.05E-05 | 1.36E-05 | 2385.6943 | 2.51E-03 |
| Kdm5b | -10.2417 | 0.813 | 3.4207 | 4.09E-05 | 1.77E-05 | 2793.8488 | 3.05E-03 |
| Pou2f1 | -10.6466 | 0.98 | 4.1355 | 9.01E-05 | 1.98E-05 | 2408.7022 | 3.35E-03 |
| Matn2 | -10.738 | 1.1242 | 0.2826 | 3.11E-05 | 2.22E-05 | 2428.9485 | 3.63E-03 |
| Insr | -10.5026 | 0.9189 | 2.0363 | 3.57E-05 | 2.27E-05 | 2602.4958 | 3.68E-03 |
| Csnk1g3 | -9.9774 | 0.7 | 8.7619 | 1.37E-02 | 2.75E-05 | 3011.2309 | 4.14E-03 |
| G3bp2 | -10.0296 | 0.7099 | 6.7698 | 2.40E-05 | 2.95E-05 | 2979.3657 | 4.35E-03 |
| Tlk2 | -10.2761 | 0.804 | 3.2147 | 2.13E-05 | 3.04E-05 | 2715.7473 | 4.46E-03 |
| Dennd4c | -10.1093 | 0.8373 | 3.1187 | 3.98E-02 | 3.54E-05 | 3083.1892 | 4.96E-03 |
| Creb3l2 | -9.7201 | 0.7272 | 3.7087 | 8.25E-02 | 3.96E-05 | 3633.6731 | 5.28E-03 |
| Tshz3 | -10.1449 | 0.739 | 3.1904 | 3.80E-05 | 4.62E-05 | 2841.7856 | 5.88E-03 |
| Nlk | -10.4242 | 0.8548 | 1.4126 | 3.78E-05 | 4.91E-05 | 2592.7975 | 6.10E-03 |
| Trabd2b | -11.8958 | 1.9617 | 0.2965 | 2.28E-01 | 5.36E-05 | 2109.9989 | 6.52E-03 |
| Pdlim4 | -10.1842 | 0.75 | 2.0264 | 3.44E-05 | 5.46E-05 | 2720.3569 | 6.59E-03 |
| Fgd5 | -11.4683 | 1.6298 | 0.9704 | 2.05E-01 | 5.89E-05 | 2302.1267 | 7.05E-03 |
| Myoc | -11.2976 | 1.8002 | 0.0312 | 7.01E-05 | 6.05E-05 | 1485.7066 | 7.20E-03 |
| Hectd4 | -10.9339 | 1.055 | 8.4818 | 3.15E-05 | 6.19E-05 | 2028.3943 | 7.31E-03 |
| Gm10125 | -11.4442 | 1.3808 | 0.6395 | 3.90E-05 | 6.58E-05 | 1864.4436 | 7.72E-03 |
| Zmym2 | -10.1154 | 0.7057 | 3.7801 | 2.99E-05 | 7.64E-05 | 2860.2696 | 8.84E-03 |
| Dop1b | -12.032 | 1.7859 | 3.7786 | 3.29E-05 | 7.77E-05 | 1604.5663 | 8.92E-03 |
| Acvr1 | -10.2774 | 0.7606 | 3.5165 | 3.59E-05 | 7.93E-05 | 2680.5406 | 8.93E-03 |
| Dnajc13 | -10.4639 | 0.8598 | 6.1451 | 2.53E-02 | 8.01E-05 | 2512.5002 | 8.95E-03 |
| Chm | -10.8098 | 0.98 | 4.3158 | 5.48E-05 | 8.14E-05 | 2180.2088 | 9.03E-03 |
| Sparcl1 | -10.1182 | 1.0653 | 0.1639 | 1.50E-01 | 8.20E-05 | 3066.6461 | 9.04E-03 |
| Sacm1l | -10.3088 | 0.757 | 7.9843 | 9.70E-05 | 9.92E-05 | 2568.4062 | 1.05E-02 |
| Arrb1 | -10.8684 | 0.9954 | 4.6147 | 3.00E-05 | 1.02E-04 | 2188.6169 | 1.05E-02 |
| Slc10a6 | -10.3223 | 0.8464 | 0.4016 | 4.42E-05 | 1.04E-04 | 2702.0686 | 1.05E-02 |
| Ap3d1 | -10.8079 | 0.9586 | 129.3556 | 1.94E-14 | 1.05E-04 | NA | 1.05E-02 |
| Dicer1 | -10.643 | 0.8895 | 4.3507 | 3.30E-05 | 1.10E-04 | 2294.5008 | 1.09E-02 |
| Rabl6 | -10.6496 | 0.8906 | 3.6543 | 3.02E-05 | 1.10E-04 | 2297.6298 | 1.09E-02 |

**Fibroblast: gene expression, negative correlation (β_1_) with age**

| **gene** | **Beta0** | **Beta1** | **Disp** | **Sigma** | **P.Beta** | **AIC** | **fdrP** |
| --- | --- | --- | --- | --- | --- | --- | --- |
| Ttll12 | -8.6382 | -2.6566 | 0.0508 | 8.62E-05 | 4.16E-23 | 1049.425 | 6.87E-19 |
| Itm2a | -9.3468 | -1.4885 | 0.2626 | 4.56E-05 | 9.30E-16 | 1478.323 | 7.69E-12 |
| Dmac2l | -9.8757 | -1.6895 | 0.3593 | 6.85E-05 | 1.73E-15 | 908.7069 | 7.96E-12 |
| Cpox | -9.4389 | -1.2957 | 0.5889 | 5.05E-05 | 1.92E-15 | 1527.788 | 7.96E-12 |
| Slco2a1 | -9.803 | -2.1622 | 0.0708 | 8.67E-04 | 2.39E-13 | 711.7543 | 7.90E-10 |
| H4f16 | -10.692 | -2.7873 | 0.1687 | 6.66E-05 | 3.91E-12 | 307.1085 | 9.24E-09 |
| Nudt1 | -9.6817 | -2.2645 | 0.043 | 4.53E-05 | 6.75E-12 | 657.9736 | 1.40E-08 |
| Fnbp1l | -9.6907 | -1.6372 | 0.0915 | 5.73E-05 | 6.18E-11 | 964.9275 | 1.14E-07 |
| 1700003G18Rik | -10.274 | -3.1133 | 0.0205 | 1.01E-04 | 4.43E-10 | 290.3885 | 6.66E-07 |
| Bphl | -9.6899 | -0.9612 | 78.086 | 6.41E-08 | 6.25E-10 | 1520.678 | 8.61E-07 |
| Tnfrsf12a | -9.0814 | -0.7681 | 1.0615 | 7.69E-05 | 9.07E-10 | 2501.312 | 1.15E-06 |
| Osgep | -9.1346 | -0.8463 | 1.0297 | 4.66E-02 | 1.28E-09 | 2336.160 | 1.51E-06 |
| Cdh18 | -10.493 | -2.0258 | 0.1079 | 6.55E-05 | 2.09E-09 | 484.2409 | 2.31E-06 |
| A2ml1 | -11.174 | -2.8484 | 0.4034 | 4.56E-05 | 4.78E-09 | 204.1599 | 4.70E-06 |
| Grid2 | -10.422 | -1.3774 | 3524199.86 | 4.31E-12 | 4.83E-09 | NA | 4.70E-06 |
| Dpagt1 | -9.6713 | -1.1054 | 0.2521 | 4.55E-05 | 5.15E-09 | 1378.401 | 4.70E-06 |
| Gm45510 | -11.243 | -2.6514 | 4545327.03 | 5.44E-05 | 1.16E-08 | 213.7092 | 9.42E-06 |
| Otx2os1 | -10.851 | -3.5019 | 0.0322 | 7.17E-05 | 1.20E-08 | 190.442 | 9.42E-06 |
| Ptprn | -10.470 | -4.4676 | 1.3723 | 3.40E-01 | 1.48E-08 | 264.7124 | 1.11E-05 |
| Pik3c2a | -8.9811 | -0.6972 | 1.3703 | 2.54E-02 | 1.69E-08 | 2764.118 | 1.19E-05 |
| Ercc4 | -10.015 | -1.0887 | 2.8428 | 3.24E-02 | 1.73E-08 | 1112.549 | 1.19E-05 |
| Slc9a3r1 | -10.168 | -1.5825 | 0.3318 | 7.78E-02 | 1.93E-08 | 771.8676 | 1.28E-05 |
| 4930512B01Rik | -10.497 | -2.8067 | 0.0196 | 6.20E-05 | 4.77E-08 | 280.4491 | 3.04E-05 |
| Cldn10 | -10.447 | -1.3724 | 0.5746 | 3.37E-05 | 5.86E-08 | 729.5842 | 3.52E-05 |
| Larp7 | -9.5924 | -0.8176 | 1.8224 | 2.60E-05 | 9.39E-08 | 1790.066 | 5.01E-05 |
| Dstyk | -9.1945 | -0.7301 | 0.7726 | 7.15E-05 | 9.40E-08 | 2368.988 | 5.01E-05 |
| Gap43 | -10.451 | -2.0713 | 0.0797 | 1.83E-01 | 1.06E-07 | 474.3637 | 5.37E-05 |
| Nwd2 | -11.443 | -2.7384 | 1311581.877 | 2.09E-46 | 2.04E-07 | 169.6892 | 9.37E-05 |
| Gps2 | -9.4373 | -0.6985 | 11.2547 | 3.52E-05 | 2.23E-07 | 2097.352 | 9.96E-05 |
| Rpl37-ps1 | -11.443 | -3.4315 | 2786497.26 | 9.92E-05 | 2.64E-07 | 132.4164 | 1.15E-04 |
| Tmprss9 | -11.356 | -2.5308 | 0.2295 | 4.03E-05 | 2.74E-07 | 200.4893 | 1.16E-04 |
| Tmem108 | -10.414 | -2.8558 | 0.0133 | 1.72E-04 | 3.22E-07 | 269.5217 | 1.33E-04 |
| Ank3 | -9.5384 | -1.8611 | 0.0252 | 1.22E-04 | 4.84E-07 | 744.6009 | 1.95E-04 |
| Tulp2 | -11.075 | -1.9082 | 0.1366 | 6.05E-05 | 1.10E-06 | 323.7713 | 4.05E-04 |
| Dcps | -9.986 | -1.0185 | 0.3151 | 5.73E-04 | 1.36E-06 | 1193.286 | 4.69E-04 |
| Cgref1 | -11.134 | -1.9863 | 0.1283 | 4.50E-05 | 1.52E-06 | 307.8059 | 5.13E-04 |
| Gm26555 | -11.175 | -2.1371 | 0.0824 | 1.34E-04 | 1.60E-06 | 268.7858 | 5.31E-04 |
| Endog | -10.144 | -0.9316 | 3476670.23 | 6.96E-26 | 1.64E-06 | 1144.791 | 5.31E-04 |
| Gm15567 | -11.382 | -3.1842 | 0.0365 | 7.47E-05 | 1.81E-06 | 148.91 | 5.75E-04 |
| Dcc | -11.561 | -2.4665 | 1758633.03 | 3.83E-15 | 1.88E-06 | 185.0384 | 5.87E-04 |
| Gm14798 | -10.934 | -1.5757 | 0.306 | 3.21E-02 | 2.48E-06 | 435.8414 | 7.59E-04 |
| Ccdc171 | -9.3632 | -0.7073 | 0.5999 | 9.61E-05 | 2.90E-06 | 2205.983 | 8.48E-04 |
| Ezh2 | -9.793 | -0.9919 | 0.4369 | 9.12E-02 | 2.99E-06 | 1404.069 | 8.48E-04 |
| Nedd9 | -8.8019 | -1.049 | 0.2528 | 1.43E-01 | 3.00E-06 | 2529.770 | 8.48E-04 |
| Dhrs7b | -9.8435 | -0.8112 | 1.2263 | 3.02E-05 | 3.06E-06 | 1490.822 | 8.48E-04 |
| 1700112J16Rik | -11.561 | -2.3329 | 6895532.53 | 3.25E-18 | 3.07E-06 | 185.0453 | 8.48E-04 |
| Ak7 | -11.443 | -2.0452 | 2300573.49 | 1.73E-15 | 3.51E-06 | 235.0312 | 9.39E-04 |
| Sumf2 | -10.056 | -0.8676 | 4.6224 | 5.86E-05 | 3.55E-06 | 1254.0842 | 9.39E-04 |
| Acd | -10.307 | -1.0095 | 1.7319 | 4.17E-05 | 3.58E-06 | 982.9097 | 9.39E-04 |
| Fzd5 | -10.297 | -1.1468 | 1.0125 | 1.03E-01 | 4.04E-06 | 908.0828 | 9.98E-04 |

**Smooth Muscle Cell: gene expression, positive correlation (β_1_) with age**

| **gene** | **Beta0** | **Beta1** | **Disp** | **Sigma** | **P.Beta** | **AIC** | **fdrP** |
| --- | --- | --- | --- | --- | --- | --- | --- |
| Hsph1 | -10.0942 | 1.1491 | 4.5698 | 1.76E-05 | 6.57E-18 | 9213.3985 | 3.86E-15 |
| Mmp3 | -11.4854 | 2.4229 | 0.1109 | 1.14E-02 | 4.91E-16 | 6658.2179 | 2.04E-13 |
| Pawr | -9.2407 | 0.7762 | 1.899 | 2.74E-02 | 4.20E-15 | 11976.2017 | 1.52E-12 |
| Ccn2 | -7.1986 | 0.9221 | 2.0758 | 1.15E-01 | 6.38E-14 | 26112.9533 | 1.84E-11 |
| Rgs17 | -11.3877 | 1.9747 | 0.255 | 4.90E-02 | 6.95E-13 | 6816.0351 | 1.74E-10 |
| Kifap3 | -10.7697 | 1.2681 | 4.9598 | 1.07E-04 | 7.61E-12 | 6930.7296 | 1.58E-09 |
| Dnah7a | -11.4622 | 1.745 | 1.5384 | 1.72E-02 | 3.96E-11 | 6140.9425 | 6.96E-09 |
| Pcnx | -10.1104 | 0.8664 | 5.8275 | 2.40E-05 | 1.11E-10 | 7952.8409 | 1.81E-08 |
| Agfg1 | -10.5882 | 1.0804 | 9.0228 | 2.17E-02 | 2.78E-10 | 6875.1303 | 3.97E-08 |
| Rpa1 | -10.7716 | 1.1717 | 2.1219 | 2.93E-05 | 3.82E-10 | 6691.0415 | 5.30E-08 |
| Arhgap32 | -11.0753 | 1.3583 | 1.3214 | 2.22E-05 | 5.40E-10 | 6262.4913 | 7.24E-08 |
| Skap2 | -9.9663 | 0.7704 | 4.3267 | 9.37E-05 | 9.35E-10 | 8249.8697 | 1.19E-07 |
| Wdr33 | -10.0601 | 0.7994 | 6.5829 | 7.19E-05 | 1.03E-09 | 7947.7933 | 1.30E-07 |
| Tmem164 | -10.4833 | 0.9962 | 1.83 | 2.58E-05 | 1.17E-09 | 7160.9317 | 1.46E-07 |
| Acsl1 | -9.9953 | 0.8749 | 3.6813 | 3.52E-02 | 1.30E-09 | 8646.1828 | 1.58E-07 |
| Taok3 | -11.397 | 1.5326 | 3.6343 | 2.96E-05 | 1.33E-09 | 5636.9627 | 1.59E-07 |
| Pdgfrb | -9.528 | 0.6938 | 17.0688 | 4.86E-02 | 1.66E-09 | 9616.1365 | 1.89E-07 |
| Me1 | -10.5331 | 0.9895 | 7.0019 | 2.51E-05 | 1.95E-09 | 6800.1894 | 2.18E-07 |
| Med1 | -11.2807 | 1.4206 | 10.1408 | 2.58E-05 | 2.28E-09 | 5614.313 | 2.51E-07 |
| C1s1 | -10.963 | 1.4497 | 0.8784 | 1.11E-01 | 2.96E-09 | 7209.8645 | 3.16E-07 |
| Tlk2 | -10.451 | 0.958 | 4.7638 | 2.99E-02 | 4.57E-09 | 7045.1912 | 4.61E-07 |
| Wscd2 | -10.4549 | 0.9353 | 3.8488 | 2.83E-05 | 4.78E-09 | 6975.1837 | 4.79E-07 |
| Numb | -10.8752 | 1.1453 | 3.8529 | 4.27E-05 | 4.82E-09 | 6157.379 | 4.81E-07 |
| Rad21 | -10.3644 | 0.8741 | 4843269.996 | 1.32E-19 | 6.62E-09 | NA | 6.48E-07 |
| Sdc2 | -8.993 | 0.8364 | 3.8016 | 9.48E-02 | 7.90E-09 | 13537.4223 | 7.65E-07 |
| St3gal3 | -11.6819 | 1.6696 | 1.5939 | 2.46E-05 | 1.17E-08 | 5211.8994 | 1.11E-06 |
| Nsmce2 | -10.1842 | 0.8072 | 1.8127 | 2.97E-05 | 1.44E-08 | 7641.883 | 1.34E-06 |
| Fam91a1 | -11.1267 | 1.2412 | 29.0392 | 2.54E-04 | 1.70E-08 | 5553.7461 | 1.54E-06 |
| Topors | -10.951 | 1.1656 | 4.274 | 2.15E-02 | 2.13E-08 | 5991.8156 | 1.90E-06 |
| Prpf19 | -10.6447 | 0.9584 | 2960181.737 | 8.73E-41 | 3.15E-08 | NA | 2.68E-06 |
| Zcchc14 | -9.8908 | 0.707 | 8.9349 | 3.19E-02 | 4.50E-08 | 8249.36 | 3.67E-06 |
| Invs | -11.175 | 1.2516 | 1.1614 | 3.91E-05 | 5.18E-08 | 5547.4978 | 4.16E-06 |
| Ptpn9 | -10.3852 | 0.8404 | 4.0787 | 2.14E-05 | 5.23E-08 | 6881.0554 | 4.18E-06 |
| Samd9l | -11.1745 | 1.334 | 2.3888 | 7.89E-02 | 5.53E-08 | 5685.486 | 4.36E-06 |
| Vwa5a | -11.0355 | 1.1384 | 25.7236 | 1.85E-05 | 6.35E-08 | 5529.6679 | 4.92E-06 |
| Ubtf | -10.3429 | 0.8068 | 17.8244 | 3.71E-05 | 6.95E-08 | 6866.3722 | 5.34E-06 |
| Zyg11b | -10.364 | 0.814 | 11.2453 | 2.17E-05 | 7.76E-08 | 6772.2462 | 5.88E-06 |
| Phc3 | -10.0603 | 0.7046 | 5.3029 | 3.51E-05 | 8.30E-08 | 7614.5226 | 6.23E-06 |
| Zhx2 | -10.5085 | 0.8784 | 3.3328 | 2.33E-05 | 8.66E-08 | 6558.7874 | 6.44E-06 |
| Slc7a2 | -10.9904 | 1.116 | 1.8805 | 3.64E-05 | 9.29E-08 | 5749.0497 | 6.85E-06 |
| Appl1 | -10.0957 | 0.7124 | 5.7761 | 2.36E-05 | 9.35E-08 | 7464.5391 | 6.87E-06 |
| Slc4a3 | -10.3646 | 0.8064 | 18.2961 | 2.12E-05 | 9.74E-08 | 6772.4809 | 7.13E-06 |
| Lin52 | -11.6056 | 1.4952 | 1.3866 | 2.62E-05 | 1.15E-07 | 4911.9606 | 8.23E-06 |
| Ttc39b | -11.081 | 1.1471 | 3.6207 | 2.58E-05 | 1.17E-07 | 5472.6274 | 8.33E-06 |
| Plekhh2 | -11.3914 | 1.3883 | 0.8113 | 2.39E-02 | 1.71E-07 | 5250.9805 | 1.16E-05 |
| Lima1 | -10.7372 | 0.9773 | 0.76 | 2.82E-05 | 1.82E-07 | 6149.0762 | 1.23E-05 |
| Brd3 | -10.5332 | 0.8603 | 6.2492 | 2.24E-05 | 1.91E-07 | 6393.391 | 1.28E-05 |
| Rps6ka5 | -11.4002 | 1.4333 | 1.6473 | 8.11E-02 | 2.19E-07 | 5544.9222 | 1.45E-05 |
| Dstyk | -10.993 | 1.0692 | 11.9833 | 2.87E-05 | 2.23E-07 | 5412.9481 | 1.46E-05 |
| Prickle2 | -11.6072 | 1.4625 | 1.2772 | 3.14E-05 | 2.24E-07 | 4848.4971 | 1.46E-05 |

**Smooth Muscle Cell: gene expression, negative correlation (β_1_) with age**

| **gene** | **Beta0** | **Beta1** | **Disp** | **Sigma** | **P.Beta** | **AIC** | **fdrP** |
| --- | --- | --- | --- | --- | --- | --- | --- |
| Ercc4 | -9.1096 | -1.9886 | 29.9402 | 1.02E-06 | 3.80E-99 | 3217.440 | 6.47E-95 |
| Sdk2 | -8.877 | -1.7655 | 0.947 | 1.60E-04 | 1.11E-76 | 4308.432 | 9.43E-73 |
| Dync1i1 | -10.2585 | -4.03 | 29.6169 | 8.87E-05 | 1.30E-46 | 572.9016 | 7.39E-43 |
| Cpox | -9.3722 | -1.561 | 0.6554 | 3.62E-05 | 8.14E-41 | 3450.984 | 3.47E-37 |
| Bex3 | -8.8533 | -0.9944 | 4.1559 | 3.34E-05 | 1.42E-36 | 6495.329 | 4.03E-33 |
| Ubb-ps | -10.5286 | -2.8321 | 2.3085 | 2.86E-05 | 3.03E-36 | 750.8064 | 7.37E-33 |
| Tpcn2 | -9.219 | -1.8777 | 0.1761 | 3.91E-05 | 3.89E-36 | 3016.925 | 8.28E-33 |
| Gap43 | -10.5615 | -3.38 | 0.2612 | 3.64E-05 | 7.45E-34 | 562.0157 | 1.41E-30 |
| Gm45609 | -10.6352 | -3.7133 | 0.2288 | 4.06E-05 | 2.38E-31 | 466.4338 | 4.05E-28 |
| Pfkfb4 | -10.0129 | -1.6925 | 0.664 | 2.61E-05 | 1.30E-28 | 2047.793 | 1.85E-25 |
| Ttll12 | -9.8035 | -2.0581 | 0.1174 | 4.63E-05 | 4.79E-27 | 1811.1 | 6.26E-24 |
| Snord104 | -10.4143 | -2.2396 | 0.2928 | 3.52E-05 | 5.14E-27 | 1111.947 | 6.26E-24 |
| Gfod2 | -9.6023 | -1.8147 | 0.1162 | 3.56E-05 | 1.32E-25 | 2338.706 | 1.50E-22 |
| Spa17 | -10.2059 | -1.667 | 1.1488 | 3.86E-05 | 8.05E-25 | 1829.469 | 8.57E-22 |
| Rny1 | -10.9479 | -3.6121 | 0.2385 | 4.04E-05 | 3.57E-24 | 373.8124 | 3.58E-21 |
| Dmac2l | -10.1428 | -1.5821 | 0.8129 | 8.17E-05 | 1.70E-23 | 1996.135 | 1.61E-20 |
| Slc2a3 | -10.0127 | -2.0805 | 0.0956 | 6.47E-05 | 1.98E-22 | 1529.567 | 1.78E-19 |
| Racgap1 | -11.1265 | -3.798 | 147746.415 | 1.94E-04 | 1.53E-21 | 304.1932 | 1.24E-18 |
| H4c14 | -10.8557 | -2.5031 | 0.2636 | 3.45E-05 | 2.02E-21 | 684.8197 | 1.57E-18 |
| Il12rb1 | -10.996 | -4.1804 | 0.2067 | 3.94E-05 | 3.60E-21 | 299.7066 | 2.67E-18 |
| Gm50455 | -11.0781 | -2.7103 | 1.1999 | 7.78E-07 | 5.66E-21 | 523.2945 | 4.02E-18 |
| Ptprn | -10.4292 | -2.3117 | 2.1372 | 1.12E-01 | 3.03E-20 | 1069.805 | 1.98E-17 |
| Dennd6b | -10.0497 | -2.3532 | 0.0477 | 4.11E-05 | 1.17E-19 | 1169.102 | 7.36E-17 |
| Gm6787 | -11.1505 | -3.4806 | 0.1085 | 4.06E-05 | 5.01E-19 | 336.8781 | 3.05E-16 |
| Batf | -11.2746 | -3.5438 | 0.1701 | 3.92E-05 | 7.42E-18 | 301.4315 | 4.22E-15 |
| H3c6 | -11.3447 | -3.1385 | 0.6863 | 3.44E-05 | 9.77E-18 | 331.6523 | 5.37E-15 |
| Mbd4 | -10.3323 | -1.7395 | 0.1399 | 3.93E-05 | 1.25E-17 | 1516.636 | 6.68E-15 |
| Slc7a14 | -11.2068 | -4.1248 | 0.1603 | 3.49E-05 | 1.52E-17 | 259.5425 | 7.87E-15 |
| Slc9a3r1 | -9.867 | -1.0749 | 2.3704 | 2.76E-05 | 7.30E-17 | 3238.361 | 3.55E-14 |
| Pcbd2 | -8.6217 | -1.1767 | 1.1261 | 6.74E-02 | 1.95E-16 | 6735.580 | 8.97E-14 |
| Gm45442 | -11.463 | -3.4615 | 2924086.52 | 9.44E-05 | 2.22E-16 | 268.1609 | 9.94E-14 |
| Dlgap2 | -11.3282 | -2.749 | 0.4224 | 3.83E-05 | 2.40E-16 | 420.1169 | 1.05E-13 |
| Mrln | -11.2782 | -2.5101 | 0.6924 | 3.65E-04 | 3.51E-16 | 490.0011 | 1.49E-13 |
| Zfp935 | -11.464 | -3.2581 | 0.3126 | 3.80E-05 | 1.10E-15 | 292.9145 | 4.46E-13 |
| 4921531C22Rik | -10.7452 | -1.6833 | 0.744 | 4.87E-05 | 1.74E-15 | 1198.277 | 6.88E-13 |
| Ddb2 | -9.826 | -1.374 | 0.1846 | 4.46E-05 | 3.78E-15 | 2706.212 | 1.40E-12 |
| H4c1 | -11.3975 | -2.6327 | 0.4857 | 4.41E-05 | 4.68E-15 | 415.6648 | 1.66E-12 |
| 1700064H15Rik | -11.1019 | -3.368 | 0.0336 | 3.95E-05 | 4.82E-15 | 345.3293 | 1.66E-12 |
| Gm48943 | -11.1753 | -4.8478 | 5766350.8 | 6.86E-05 | 4.88E-15 | NA | 1.66E-12 |
| Fhod3 | -10.3456 | -1.7814 | 0.0769 | 7.78E-05 | 1.12E-14 | 1391.297 | 3.74E-12 |
| 4930513L16Rik | -11.4094 | -4.3256 | 0.5193 | 3.85E-05 | 1.63E-14 | 209.7112 | 5.33E-12 |
| Ticrr | -11.5337 | -3.1013 | 0.2725 | 3.20E-05 | 1.76E-14 | 299.0011 | 5.67E-12 |
| Plxdc1 | -9.6222 | -4.8768 | 0.0062 | 8.85E-05 | 1.94E-14 | 318.1192 | 6.13E-12 |
| U2af1 | -9.4348 | -0.7545 | 450.3833 | 2.16E-13 | 2.18E-14 | 5161.631 | 6.75E-12 |
| Znrf2 | -9.6563 | -0.8895 | 1.8 | 3.82E-05 | 2.43E-14 | 4180.499 | 7.31E-12 |
| Edn1 | -10.9649 | -2.5304 | 0.0492 | 4.79E-05 | 2.45E-14 | 584.179 | 7.31E-12 |
| Fgd1 | -9.8162 | -1.0077 | 0.7964 | 3.18E-05 | 2.53E-14 | 3534.584 | 7.43E-12 |
| Gm27151 | -11.039 | -1.8923 | 0.462 | 4.28E-05 | 6.90E-14 | 837.5217 | 1.96E-11 |
| AC113970.1 | -11.4556 | -4.2791 | 0.2379 | 3.95E-05 | 7.11E-14 | 208.296 | 1.99E-11 |
| Uckl1 | -9.4214 | -1.2288 | 1.3454 | 9.01E-02 | 1.01E-13 | 4023.754 | 2.78E-11 |

**Myeloid: gene expression, positive correlation (β_1_) with age**

| **gene** | **Beta0** | **Beta1** | **Disp** | **Sigma** | **P.Beta** | **AIC** | **fdrP** |
| --- | --- | --- | --- | --- | --- | --- | --- |
| Itih5 | -9.2953 | 0.731 | 0.7305 | 7.02E-05 | 5.97E-08 | 4587.9208 | 3.52E-05 |
| Vps13d | -10.4628 | 1.1157 | 8.1092 | 2.70E-05 | 8.71E-08 | 2858.1634 | 4.97E-05 |
| C130026I21Rik | -10.1638 | 1.2109 | 1.2616 | 1.22E-01 | 1.21E-07 | 3724.8345 | 5.87E-05 |
| Prelp | -9.1327 | 0.7997 | 1.6873 | 1.00E-01 | 1.50E-07 | 5210.2008 | 7.07E-05 |
| Pknox2 | -11.4461 | 1.705 | 1.2443 | 6.29E-05 | 5.65E-07 | 2279.0031 | 2.22E-04 |
| Kdm2a | -9.7268 | 0.7347 | 4.9688 | 1.94E-02 | 1.22E-06 | 3565.9903 | 4.28E-04 |
| Mllt10 | -9.5271 | 0.7147 | 4.5736 | 6.15E-02 | 3.08E-06 | 3951.8103 | 8.48E-04 |
| Mdm4 | -10.8094 | 1.1451 | 4.4466 | 3.32E-05 | 3.89E-06 | 2400.4257 | 9.98E-04 |
| Trim8 | -10.384 | 0.93 | 4.7436 | 4.20E-05 | 4.07E-06 | 2736.8369 | 9.98E-04 |
| Ngf | -10.5408 | 1.0882 | 0.4094 | 6.06E-05 | 4.15E-06 | 2739.5772 | 9.98E-04 |
| Lipa | -10.5112 | 0.9936 | 2.5004 | 5.12E-05 | 4.16E-06 | 2659.9914 | 9.98E-04 |
| Klhl29 | -10.0326 | 0.8381 | 0.7139 | 3.16E-05 | 4.36E-06 | 3236.8469 | 1.03E-03 |
| Rab7b | -11.0042 | 1.2496 | 3.138 | 2.85E-05 | 4.78E-06 | 2159.1945 | 1.11E-03 |
| Gypc | -11.8501 | 1.8889 | 10.4464 | 2.00E-02 | 5.37E-06 | 1928.2471 | 1.23E-03 |
| Ilrun | -10.55 | 0.9862 | 663.4363 | 3.54E-14 | 5.53E-06 | 2526.967 | 1.25E-03 |
| Map2k5 | -11.095 | 1.3961 | 3.7014 | 1.09E-01 | 5.77E-06 | 2406.5062 | 1.29E-03 |
| Uba6 | -10.8086 | 1.1133 | 4.7893 | 3.33E-05 | 7.14E-06 | 2307.3531 | 1.55E-03 |
| Ncoa1 | -9.3136 | 0.6958 | 2.7014 | 8.82E-02 | 9.06E-06 | 4525.6458 | 1.85E-03 |
| Zfp950 | -10.7511 | 1.1781 | 2.259 | 6.62E-02 | 9.24E-06 | 2577.1546 | 1.86E-03 |
| Zscan26 | -11.3383 | 1.4025 | 14.191 | 1.55E-03 | 1.20E-05 | 2014.4047 | 2.26E-03 |
| Bmp6 | -10.765 | 1.1766 | 0.2241 | 5.05E-05 | 1.36E-05 | 2385.6943 | 2.51E-03 |
| Kdm5b | -10.2417 | 0.813 | 3.4207 | 4.09E-05 | 1.77E-05 | 2793.8488 | 3.05E-03 |
| Pou2f1 | -10.6466 | 0.98 | 4.1355 | 9.01E-05 | 1.98E-05 | 2408.7022 | 3.35E-03 |
| Matn2 | -10.738 | 1.1242 | 0.2826 | 3.11E-05 | 2.22E-05 | 2428.9485 | 3.63E-03 |
| Insr | -10.5026 | 0.9189 | 2.0363 | 3.57E-05 | 2.27E-05 | 2602.4958 | 3.68E-03 |
| Csnk1g3 | -9.9774 | 0.7 | 8.7619 | 1.37E-02 | 2.75E-05 | 3011.2309 | 4.14E-03 |
| G3bp2 | -10.0296 | 0.7099 | 6.7698 | 2.40E-05 | 2.95E-05 | 2979.3657 | 4.35E-03 |
| Tlk2 | -10.2761 | 0.804 | 3.2147 | 2.13E-05 | 3.04E-05 | 2715.7473 | 4.46E-03 |
| Dennd4c | -10.1093 | 0.8373 | 3.1187 | 3.98E-02 | 3.54E-05 | 3083.1892 | 4.96E-03 |
| Creb3l2 | -9.7201 | 0.7272 | 3.7087 | 8.25E-02 | 3.96E-05 | 3633.6731 | 5.28E-03 |
| Tshz3 | -10.1449 | 0.739 | 3.1904 | 3.80E-05 | 4.62E-05 | 2841.7856 | 5.88E-03 |
| Nlk | -10.4242 | 0.8548 | 1.4126 | 3.78E-05 | 4.91E-05 | 2592.7975 | 6.10E-03 |
| Trabd2b | -11.8958 | 1.9617 | 0.2965 | 2.28E-01 | 5.36E-05 | 2109.9989 | 6.52E-03 |
| Pdlim4 | -10.1842 | 0.75 | 2.0264 | 3.44E-05 | 5.46E-05 | 2720.3569 | 6.59E-03 |
| Fgd5 | -11.4683 | 1.6298 | 0.9704 | 2.05E-01 | 5.89E-05 | 2302.1267 | 7.05E-03 |
| Myoc | -11.2976 | 1.8002 | 0.0312 | 7.01E-05 | 6.05E-05 | 1485.7066 | 7.20E-03 |
| Hectd4 | -10.9339 | 1.055 | 8.4818 | 3.15E-05 | 6.19E-05 | 2028.3943 | 7.31E-03 |
| Gm10125 | -11.4442 | 1.3808 | 0.6395 | 3.90E-05 | 6.58E-05 | 1864.4436 | 7.72E-03 |
| Zmym2 | -10.1154 | 0.7057 | 3.7801 | 2.99E-05 | 7.64E-05 | 2860.2696 | 8.84E-03 |
| Dop1b | -12.032 | 1.7859 | 3.7786 | 3.29E-05 | 7.77E-05 | 1604.5663 | 8.92E-03 |
| Acvr1 | -10.2774 | 0.7606 | 3.5165 | 3.59E-05 | 7.93E-05 | 2680.5406 | 8.93E-03 |
| Dnajc13 | -10.4639 | 0.8598 | 6.1451 | 2.53E-02 | 8.01E-05 | 2512.5002 | 8.95E-03 |
| Chm | -10.8098 | 0.98 | 4.3158 | 5.48E-05 | 8.14E-05 | 2180.2088 | 9.03E-03 |
| Sparcl1 | -10.1182 | 1.0653 | 0.1639 | 1.50E-01 | 8.20E-05 | 3066.6461 | 9.04E-03 |
| Sacm1l | -10.3088 | 0.757 | 7.9843 | 9.70E-05 | 9.92E-05 | 2568.4062 | 1.05E-02 |
| Arrb1 | -10.8684 | 0.9954 | 4.6147 | 3.00E-05 | 1.02E-04 | 2188.6169 | 1.05E-02 |
| Slc10a6 | -10.3223 | 0.8464 | 0.4016 | 4.42E-05 | 1.04E-04 | 2702.0686 | 1.05E-02 |
| Ap3d1 | -10.8079 | 0.9586 | 129.3556 | 1.94E-14 | 1.05E-04 | NA | 1.05E-02 |
| Dicer1 | -10.643 | 0.8895 | 4.3507 | 3.30E-05 | 1.10E-04 | 2294.5008 | 1.09E-02 |
| Rabl6 | -10.6496 | 0.8906 | 3.6543 | 3.02E-05 | 1.10E-04 | 2297.6298 | 1.09E-02 |

**Myeloid: gene expression, negative correlation (β_1_) with age**

| **gene** | **Beta0** | **Beta1** | **Disp** | **Sigma** | **P.Beta** | **AIC** | **fdrP** |
| --- | --- | --- | --- | --- | --- | --- | --- |
| Ttll12 | -8.6382 | -2.6566 | 0.0508 | 8.62E-05 | 4.16E-23 | 1049.425 | 6.87E-19 |
| Itm2a | -9.3468 | -1.4885 | 0.2626 | 4.56E-05 | 9.30E-16 | 1478.323 | 7.69E-12 |
| Dmac2l | -9.8757 | -1.6895 | 0.3593 | 6.85E-05 | 1.73E-15 | 908.7069 | 7.96E-12 |
| Cpox | -9.4389 | -1.2957 | 0.5889 | 5.05E-05 | 1.92E-15 | 1527.788 | 7.96E-12 |
| Slco2a1 | -9.803 | -2.1622 | 0.0708 | 8.67E-04 | 2.39E-13 | 711.7543 | 7.90E-10 |
| H4f16 | -10.6921 | -2.7873 | 0.1687 | 6.66E-05 | 3.91E-12 | 307.1085 | 9.24E-09 |
| Nudt1 | -9.6817 | -2.2645 | 0.043 | 4.53E-05 | 6.75E-12 | 657.9736 | 1.40E-08 |
| Fnbp1l | -9.6907 | -1.6372 | 0.0915 | 5.73E-05 | 6.18E-11 | 964.9275 | 1.14E-07 |
| 1700003G18Rik | -10.2742 | -3.1133 | 0.0205 | 1.01E-04 | 4.43E-10 | 290.3885 | 6.66E-07 |
| Bphl | -9.6899 | -0.9612 | 78.086 | 6.41E-08 | 6.25E-10 | 1520.678 | 8.61E-07 |
| Tnfrsf12a | -9.0814 | -0.7681 | 1.0615 | 7.69E-05 | 9.07E-10 | 2501.312 | 1.15E-06 |
| Osgep | -9.1346 | -0.8463 | 1.0297 | 4.66E-02 | 1.28E-09 | 2336.160 | 1.51E-06 |
| Cdh18 | -10.4934 | -2.0258 | 0.1079 | 6.55E-05 | 2.09E-09 | 484.2409 | 2.31E-06 |
| A2ml1 | -11.1743 | -2.8484 | 0.4034 | 4.56E-05 | 4.78E-09 | 204.1599 | 4.70E-06 |
| Grid2 | -10.4222 | -1.3774 | 3524199.86 | 4.31E-12 | 4.83E-09 | NA | 4.70E-06 |
| Dpagt1 | -9.6713 | -1.1054 | 0.2521 | 4.55E-05 | 5.15E-09 | 1378.401 | 4.70E-06 |
| Gm45510 | -11.2432 | -2.6514 | 4545327.03 | 5.44E-05 | 1.16E-08 | 213.7092 | 9.42E-06 |
| Otx2os1 | -10.8512 | -3.5019 | 0.0322 | 7.17E-05 | 1.20E-08 | 190.442 | 9.42E-06 |
| Ptprn | -10.4707 | -4.4676 | 1.3723 | 3.40E-01 | 1.48E-08 | 264.7124 | 1.11E-05 |
| Pik3c2a | -8.9811 | -0.6972 | 1.3703 | 2.54E-02 | 1.69E-08 | 2764.118 | 1.19E-05 |
| Ercc4 | -10.0155 | -1.0887 | 2.8428 | 3.24E-02 | 1.73E-08 | 1112.549 | 1.19E-05 |
| Slc9a3r1 | -10.1688 | -1.5825 | 0.3318 | 7.78E-02 | 1.93E-08 | 771.8676 | 1.28E-05 |
| 4930512B01Rik | -10.4978 | -2.8067 | 0.0196 | 6.20E-05 | 4.77E-08 | 280.4491 | 3.04E-05 |
| Cldn10 | -10.447 | -1.3724 | 0.5746 | 3.37E-05 | 5.86E-08 | 729.5842 | 3.52E-05 |
| Larp7 | -9.5924 | -0.8176 | 1.8224 | 2.60E-05 | 9.39E-08 | 1790.066 | 5.01E-05 |
| Dstyk | -9.1945 | -0.7301 | 0.7726 | 7.15E-05 | 9.40E-08 | 2368.988 | 5.01E-05 |
| Gap43 | -10.4514 | -2.0713 | 0.0797 | 1.83E-01 | 1.06E-07 | 474.3637 | 5.37E-05 |
| Nwd2 | -11.4438 | -2.7384 | 1311581.87 | 2.09E-46 | 2.04E-07 | 169.6892 | 9.37E-05 |
| Gps2 | -9.4373 | -0.6985 | 11.2547 | 3.52E-05 | 2.23E-07 | 2097.352 | 9.96E-05 |
| Rpl37-ps1 | -11.4438 | -3.4315 | 2786497.26 | 9.92E-05 | 2.64E-07 | 132.4164 | 1.15E-04 |
| Tmprss9 | -11.3566 | -2.5308 | 0.2295 | 4.03E-05 | 2.74E-07 | 200.4893 | 1.16E-04 |
| Tmem108 | -10.4145 | -2.8558 | 0.0133 | 1.72E-04 | 3.22E-07 | 269.5217 | 1.33E-04 |
| Ank3 | -9.5384 | -1.8611 | 0.0252 | 1.22E-04 | 4.84E-07 | 744.6009 | 1.95E-04 |
| Tulp2 | -11.0756 | -1.9082 | 0.1366 | 6.05E-05 | 1.10E-06 | 323.7713 | 4.05E-04 |
| Dcps | -9.986 | -1.0185 | 0.3151 | 5.73E-04 | 1.36E-06 | 1193.286 | 4.69E-04 |
| Cgref1 | -11.1348 | -1.9863 | 0.1283 | 4.50E-05 | 1.52E-06 | 307.8059 | 5.13E-04 |
| Gm26555 | -11.1753 | -2.1371 | 0.0824 | 1.34E-04 | 1.60E-06 | 268.7858 | 5.31E-04 |
| Endog | -10.1446 | -0.9316 | 3476670.23 | 6.96E-26 | 1.64E-06 | 1144.791 | 5.31E-04 |
| Gm15567 | -11.3827 | -3.1842 | 0.0365 | 7.47E-05 | 1.81E-06 | 148.91 | 5.75E-04 |
| Dcc | -11.5616 | -2.4665 | 1758633.03 | 3.83E-15 | 1.88E-06 | 185.0384 | 5.87E-04 |
| Gm14798 | -10.9343 | -1.5757 | 0.306 | 3.21E-02 | 2.48E-06 | 435.8414 | 7.59E-04 |
| Ccdc171 | -9.3632 | -0.7073 | 0.5999 | 9.61E-05 | 2.90E-06 | 2205.983 | 8.48E-04 |
| Ezh2 | -9.793 | -0.9919 | 0.4369 | 9.12E-02 | 2.99E-06 | 1404.069 | 8.48E-04 |
| Nedd9 | -8.8019 | -1.049 | 0.2528 | 1.43E-01 | 3.00E-06 | 2529.770 | 8.48E-04 |
| Dhrs7b | -9.8435 | -0.8112 | 1.2263 | 3.02E-05 | 3.06E-06 | 1490.822 | 8.48E-04 |
| 1700112J16Rik | -11.5616 | -2.3329 | 6895532.5 | 3.25E-18 | 3.07E-06 | 185.0453 | 8.48E-04 |
| Ak7 | -11.4438 | -2.0452 | 2300573.4 | 1.73E-15 | 3.51E-06 | 235.0312 | 9.39E-04 |
| Sumf2 | -10.0564 | -0.8676 | 4.6224 | 5.86E-05 | 3.55E-06 | 1254.084 | 9.39E-04 |
| Acd | -10.307 | -1.0095 | 1.7319 | 4.17E-05 | 3.58E-06 | 982.9097 | 9.39E-04 |
| Fzd5 | -10.2974 | -1.1468 | 1.0125 | 1.03E-01 | 4.04E-06 | 908.0828 | 9.98E-04 |

**Perivascular Macrophage: gene expression, positive correlation (β_1_) with age**

| **gene** | **Beta0** | **Beta1** | **Disp** | **Sigma** | **P.Beta** | **AIC** | **fdrP** |
| --- | --- | --- | --- | --- | --- | --- | --- |
| Wdr33 | -10.2491 | 1.16E+14 | 7.743034 | 4.13E-05 | 7.91E-07 | 1741.861 | 0.000803 |
| C130026I21Rik | -9.82924 | 1.36E+14 | 2.324261 | 0.300124 | 1.34E-05 | 2719.183 | 0.006198 |
| Pacs1 | -9.85837 | 8.97E+14 | 0.88233 | 3.72E-05 | 1.50E-05 | 2009.327 | 0.006533 |
| Slc23a2 | -9.99414 | 9.85E+14 | 2.399098 | 0.137919 | 5.69E-05 | 1947.387 | 0.015221 |
| Lrp10 | -10.6268 | 1.14E+14 | 8.734029 | 5.66E-05 | 6.33E-05 | 1386.645 | 0.016607 |
| Cul1 | -9.71421 | 7.52E+12 | 1.987161 | 8.52E-05 | 6.60E-05 | 2026.611 | 0.017036 |
| Gm20732 | -11.804 | 2.01E+14 | 7.727827 | 3.43E-05 | 7.04E-05 | 1111.904 | 0.017856 |
| Pde7b | -9.73319 | 1.29E+13 | 0.595869 | 0.311457 | 7.21E-05 | 2781.896 | 0.017985 |
| Mier1 | -9.4094 | 7.24E+14 | 25.16253 | 0.071992 | 0.000102 | 2293.79 | 0.022156 |
| Hs2st1 | -10.7952 | 1.18E+14 | 3.939956 | 0.000121 | 0.000128 | 1296.501 | 0.025307 |
| Oas2 | -11.8703 | 2.2E+14 | 2.190779 | 0.364483 | 0.000207 | 1418.869 | 0.033151 |
| Exoc6 | -11.2554 | 1.43E+14 | 1.169288 | 5.55E-05 | 0.000238 | 1133.538 | 0.035821 |
| Lonp2 | -11.2591 | 1.6E+14 | 6.838344 | 0.212683 | 0.000257 | 1330.589 | 0.036205 |
| Dst | -9.91393 | 8.14E+14 | 0.428516 | 4.18E-05 | 0.000259 | 1810.248 | 0.036205 |
| Ecpas | -10.1956 | 1.04E+14 | 45.21069 | 0.143044 | 0.000289 | 1762.998 | 0.038428 |
| Ifi209 | -11.2522 | 1.47E+14 | 1.448757 | 0.119754 | 0.00029 | 1180.27 | 0.038428 |
| Pnn | -10.7949 | 1.2E+14 | 6.593619 | 0.129663 | 0.000332 | 1358.656 | 0.04013 |
| R3hdm1 | -10.3014 | 8.4E+14 | 4.039403 | 2.94E-05 | 0.000588 | 1474.345 | 0.060437 |
| Slfn5 | -9.86174 | 1.05E+14 | 1.578997 | 0.279388 | 0.000594 | 2315.354 | 0.060648 |
| Maea | -10.7935 | 1.05E+14 | 13.75695 | 0.001059 | 0.00067 | 1201.315 | 0.065349 |
| Fbxo42 | -12.4969 | 2.41E+14 | 3.761251 | 5.18E-05 | 0.000724 | 900.3149 | 0.067581 |
| Cemip2 | -10.4114 | 8.88E+14 | 1.420468 | 3.89E-05 | 0.000734 | 1407.342 | 0.067848 |
| Nrf1 | -10.2068 | 7.99E+14 | 1.470844 | 5.01E-05 | 0.000742 | 1521.642 | 0.068012 |
| Acox3 | -11.4017 | 1.4E+14 | 3.706061 | 4.00E-05 | 0.000765 | 1007.529 | 0.069255 |
| Iigp1 | -10.9597 | 1.67E+14 | 0.167758 | 0.393213 | 0.000774 | 1427.614 | 0.069255 |
| Phka2 | -11.2481 | 1.3E+14 | 3.418387 | 9.14E-05 | 0.000781 | 1043.493 | 0.069529 |
| Plscr4 | -10.8061 | 1.06E+14 | 1.006683 | 3.17E-05 | 0.000815 | 1213.145 | 0.070828 |
| Parp12 | -10.8946 | 1.16E+14 | 4.28709 | 0.12357 | 0.000844 | 1242.307 | 0.071465 |
| Cxcl16 | -9.76465 | 8.85E+14 | 0.565257 | 0.189241 | 0.000864 | 2052.268 | 0.071846 |
| Rsad2 | -12.0591 | 2.03E+14 | 0.135854 | 4.74E-05 | 0.000877 | 893.9832 | 0.07257 |
| Mdfic | -9.55998 | 8.68E+13 | 3.168202 | 0.233776 | 0.00089 | 2396.548 | 0.073233 |
| Palld | -11.5827 | 1.53E+14 | 0.712142 | 3.71E-05 | 0.000909 | 960.2112 | 0.073697 |
| Fyb | -9.17789 | 1.25E+14 | 2.565098 | 0.438688 | 0.000922 | 3687.861 | 0.073697 |
| Ash1l | -9.62112 | 9.63E+14 | 24.76211 | 0.282819 | 0.001008 | 2367.463 | 0.077836 |
| Chordc1 | -10.7082 | 9.76E+14 | 2.675481 | 5.23E-05 | 0.001078 | 1219.672 | 0.081218 |
| Odr4 | -10.7056 | 9.64E+14 | 11.35319 | 0.000217 | 0.001132 | 1198.247 | 0.084068 |
| Ube2e2 | -9.98725 | 8.04E+14 | 0.524304 | 0.103711 | 0.001145 | 1753.223 | 0.084582 |
| Btaf1 | -9.42934 | 7.9E+14 | 3.14584 | 0.219364 | 0.001179 | 2281.038 | 0.086249 |
| Ccl8 | -8.71364 | 2.27E+14 | 0.306255 | 0.878004 | 0.001211 | 6144.239 | 0.087736 |
| Ncoa7 | -10.2435 | 9.32E+14 | 4.943974 | 0.168955 | 0.001293 | 1613.352 | 0.08966 |
| Pja2 | -10.3001 | 7.81E+14 | 7.241713 | 3.44E-05 | 0.00135 | 1394.965 | 0.0912 |
| Ccdc6 | -11.8048 | 1.62E+14 | 14650655 | 1.17E-22 | 0.001374 | 859.6717 | 0.0912 |
| Gpr137b-ps | -11.1113 | 1.16E+14 | 2.506798 | 7.27E-05 | 0.001376 | 1043.583 | 0.0912 |
| Ddx58 | -10.7872 | 1.06E+13 | 1.996264 | 0.097915 | 0.001395 | 1265.26 | 0.091887 |
| Tbrg1 | -10.8885 | 1.03E+14 | 9701360 | 3.29E-27 | 0.00146 | NA | 0.093738 |
| Pld1 | -10.539 | 8.95E+14 | 1.154557 | 3.44E-05 | 0.001475 | 1351.005 | 0.094314 |
| Cpq | -9.52281 | 7.39E+14 | 2.737441 | 0.185975 | 0.001497 | 2256.159 | 0.094456 |
| Csnk1g1 | -10.6458 | 1.07E+14 | 4.44131 | 0.186143 | 0.001498 | 1410.502 | 0.094456 |
| Tgs1 | -10.5494 | 8.79E+14 | 3.112608 | 8.21E-05 | 0.001504 | 1287.172 | 0.094456 |
| Arap1 | -11.2451 | 1.23E+14 | 1.724643 | 3.05E-05 | 0.001537 | 995.6132 | 0.094456 |

**Perivascular Macrophage: gene expression, negative correlation (β_1_) with age**

| **gene** | **Beta0** | **Beta1** | **Disp** | **Sigma** | **P.Beta** | **AIC** | **fdrP** |
| --- | --- | --- | --- | --- | --- | --- | --- |
| Nr1d1 | -9.76518 | -1.9E+14 | 1.120959 | 3.52E-05 | 7.49E-14 | 534.7277 | 1.14E-09 |
| Gm12236 | -10.2667 | -3.2E+14 | 0.737129 | 9.52E-05 | 1.32E-11 | 223.5961 | 1.01E-07 |
| Cdkl4 | -10.0196 | -1.8E+13 | 0.70504 | 4.98E-05 | 4.08E-10 | 469.6472 | 2.07E-06 |
| Nedd4l | -8.73245 | -9.4E+13 | 0.405558 | 4.31E-05 | 4.17E-09 | 1597.167 | 1.16E-05 |
| Cirbp | -9.50218 | -1.1E+14 | 3863530 | 1.69E-21 | 4.56E-09 | NA | 1.16E-05 |
| Als2 | -9.45906 | -1.6E+14 | 0.113399 | 9.72E-05 | 5.38E-09 | 691.7965 | 1.17E-05 |
| Tmem189 | -10.6572 | -2.6E+14 | 0.2499 | 4.81E-05 | 5.81E-08 | 199.4201 | 0.000111 |
| Rapgef4 | -9.34593 | -5.1E+14 | 0.006382 | 7.01E-05 | 1.18E-07 | 150.4907 | 0.00018 |
| Entr1 | -9.47482 | -9.5E+14 | 8.606352 | 0.006897 | 1.71E-07 | 1026.62 | 0.000236 |
| Dut | -10.3083 | -1.8E+14 | 0.192186 | 0.000146 | 2.27E-07 | 354.0351 | 0.000288 |
| Tceal8 | -9.50926 | -9.6E+14 | 2.891246 | 3.28E-05 | 2.98E-07 | 958.3001 | 0.000348 |
| Anp32e | -8.92434 | -8.3E+14 | 1.229784 | 0.076743 | 9.75E-07 | 1536.561 | 0.000928 |
| Vtcn1 | -10.2134 | -2.4E+14 | 0.037738 | 5.37E-05 | 1.07E-06 | 273.7492 | 0.00096 |
| Myo15 | -10.7384 | -2.1E+14 | 0.219962 | 0.000158 | 1.51E-06 | 230.4769 | 0.001259 |
| Rexo1 | -9.47406 | -1E+14 | 3.668727 | 0.126172 | 1.57E-06 | 996.9921 | 0.001259 |
| 6030469F06Rik | -10.5343 | -2.8E+14 | 0.276984 | 0.39388 | 2.25E-06 | 208.0938 | 0.001711 |
| Pdpn | -10.498 | -1.9E+14 | 0.136644 | 5.27E-05 | 2.88E-06 | 303.0358 | 0.002088 |
| Gm43305 | -10.6197 | -1.8E+14 | 0.513826 | 6.64E-05 | 3.21E-06 | 308.6423 | 0.002219 |
| Tmem185b | -10.0597 | -1.2E+14 | 2.230677 | 4.25E-05 | 4.00E-06 | 625.9755 | 0.002646 |
| AI506816 | -10.0127 | -1.1E+14 | 20.96745 | 8.48E-05 | 4.53E-06 | 648.7212 | 0.002874 |
| Polr2i | -9.38496 | -7.7E+14 | 8.851386 | 3.59E-08 | 5.29E-06 | 1143.206 | 0.003218 |
| Plk2 | -8.18476 | -1E+14 | 1.136623 | 0.240145 | 6.40E-06 | 2129.752 | 0.003747 |
| Mcm7 | -10.2038 | -1.4E+14 | 0.190212 | 5.39E-05 | 8.71E-06 | 470.784 | 0.004907 |
| Ndst3 | -10.2887 | -1.6E+14 | 0.092934 | 5.83E-05 | 1.01E-05 | 384.6355 | 0.00511 |
| Dnmt1 | -9.71662 | -9.1E+14 | 2.825995 | 4.62E-05 | 1.14E-05 | 884.3698 | 0.005604 |
| Ints2 | -10.057 | -1.1E+14 | 7.094496 | 0.000169 | 1.25E-05 | 626.386 | 0.005967 |
| Suclg2 | -9.48424 | -8.7E+14 | 1.210842 | 0.032862 | 1.46E-05 | 1015.87 | 0.006533 |
| Cacnb4 | -10.6336 | -1.9E+14 | 0.081794 | 4.46E-05 | 1.66E-05 | 265.2866 | 0.007036 |
| Nkain3 | -10.961 | -2.2E+13 | 0.483763 | 5.74E-05 | 2.06E-05 | 207.1612 | 0.008482 |
| Cux2 | -10.9241 | -2.2E+14 | 0.077162 | 4.82E-05 | 2.22E-05 | 185.3154 | 0.008898 |
| Gpr158 | -10.9506 | -2.1E+14 | 0.149672 | 0.000114 | 2.38E-05 | 195.9501 | 0.008905 |
| Catsper2 | -9.80053 | -2.7E+14 | 0.011431 | 6.21E-05 | 2.42E-05 | 235.2916 | 0.008905 |
| Taf1c | -10.7048 | -1.5E+14 | 2.341834 | 3.21E-05 | 2.52E-05 | 296.4804 | 0.008905 |
| Zbtb24 | -10.3578 | -1.2E+14 | 5274390 | 1.03E-06 | 2.58E-05 | 454.9864 | 0.008939 |
| Gm15787 | -10.7904 | -1.7E+14 | 0.658435 | 4.13E-05 | 2.86E-05 | 272.1116 | 0.009475 |
| Csf3 | -10.9647 | -3.6E+14 | 0.03974 | 0.000113 | 3.04E-05 | 120.7375 | 0.009737 |
| Ndufs3 | -9.07606 | -1.3E+14 | 0.203509 | 0.217726 | 3.07E-05 | 1047.024 | 0.009737 |
| Dcaf15 | -10.6261 | -1.4E+14 | 4454851 | 2.15E-05 | 3.72E-05 | 355.762 | 0.01156 |
| Shmt1 | -9.96433 | -2.1E+14 | 0.078422 | 0.214357 | 3.92E-05 | 382.49 | 0.011944 |
| Gm5586 | -10.9938 | -1.8E+14 | 2414958 | 4.84E-28 | 4.20E-05 | NA | 0.012327 |
| Mir692-1 | -10.0645 | -4.6E+14 | 0.821117 | 0.971475 | 4.21E-05 | 342.19 | 0.012327 |
| Mir320 | -10.8328 | -4.4E+14 | 0.048367 | 4.39E-05 | 4.76E-05 | 115.5813 | 0.013654 |
| Rcl1 | -10.7062 | -1.4E+14 | 6240705 | 6.13E-27 | 5.05E-05 | NA | 0.01406 |
| Il31ra | -9.85344 | -8.9E+13 | 2.746239 | 0.000145 | 5.08E-05 | 842.9278 | 0.01406 |
| Pou6f2 | -11.1474 | -3E+14 | 0.041697 | 0.000232 | 7.53E-05 | 117.9871 | 0.018475 |
| Pbdc1 | -9.38878 | -1.1E+14 | 1.537566 | 0.244164 | 9.04E-05 | 967.4087 | 0.02106 |
| Pde11a | -11.3993 | -3.2E+14 | 2611950 | 1.92E-20 | 9.22E-05 | NA | 0.02106 |
| Fam207a | -10.0451 | -9.7E+14 | 1.09593 | 3.32E-05 | 9.25E-05 | 664.1323 | 0.02106 |
| Nipsnap3b | -9.63535 | -7.6E+13 | 2.342757 | 2.75E-05 | 9.27E-05 | 971.2015 | 0.02106 |

**Endothelial Cell: gene expression, positive correlation (β_1_) with age**

| **gene** | **Beta0** | **Beta1** | **Disp** | **Sigma** | **P.Beta** | **AIC** | **fdrP** |
| --- | --- | --- | --- | --- | --- | --- | --- |
| Ltbp1 | -9.8737 | 1.0862 | 0.2587 | 9.79E-05 | 8.38E-21 | 4292.4096 | 7.33E-17 |
| Chrm3 | -10.4814 | 1.7225 | 0.1218 | 1.14E-01 | 7.52E-17 | 3677.8082 | 2.63E-13 |
| Selenop | -8.5193 | 1.0215 | 0.6718 | 1.24E-01 | 8.04E-15 | 9149.1037 | 2.34E-11 |
| Slco3a1 | -10.7175 | 1.5002 | 0.2914 | 1.67E-01 | 4.20E-11 | 3162.0626 | 4.90E-08 |
| Stbd1 | -11.5977 | 1.309 | 0.5659 | 3.54E-04 | 1.06E-09 | 1553.2323 | 1.03E-06 |
| Ubash3b | -11.7315 | 1.5603 | 0.099 | 6.80E-02 | 4.27E-09 | 1545.0672 | 3.55E-06 |
| Prelp | -11.839 | 1.9135 | 0.1696 | 2.11E-01 | 6.72E-09 | 1781.6542 | 4.97E-06 |
| Stard8 | -10.9684 | 1.0216 | 1.5107 | 4.59E-02 | 6.82E-09 | 2119.0936 | 4.97E-06 |
| Emb | -11.0889 | 1.0495 | 0.205 | 2.85E-05 | 1.34E-08 | 1901.0334 | 8.68E-06 |
| Foxo3 | -10.4778 | 0.7462 | 0.9349 | 3.81E-05 | 1.46E-08 | 2640.3614 | 9.12E-06 |
| Gpd2 | -10.5518 | 0.7664 | 0.5957 | 5.28E-05 | 3.59E-08 | 2509.3174 | 1.90E-05 |
| Sh3tc1 | -11.7102 | 1.2588 | 0.4695 | 3.26E-05 | 3.97E-08 | 1407.8605 | 2.04E-05 |
| Lin52 | -11.0312 | 0.939 | 0.4785 | 5.25E-05 | 4.47E-08 | 1920.5568 | 2.24E-05 |
| Hipk2 | -10.9572 | 0.8698 | 1.3283 | 4.35E-02 | 2.26E-07 | 1966.5143 | 8.24E-05 |
| Cdc42ep1 | -10.4267 | 0.7173 | 5.401 | 6.25E-02 | 3.21E-07 | 2631.1639 | 1.10E-04 |
| Gja1 | -11.5829 | 1.672 | 0.1116 | 2.27E-01 | 3.48E-07 | 1849.261 | 1.15E-04 |
| Akap12 | -10.9498 | 1.6299 | 0.335 | 2.86E-01 | 5.33E-07 | 2998.5377 | 1.64E-04 |
| Unc5b | -10.5635 | 0.6998 | 0.6243 | 1.82E-04 | 5.69E-07 | 2360.8947 | 1.72E-04 |
| Thy1 | -13.703 | 3.0131 | 0.0469 | 6.65E-05 | 7.61E-07 | 904.0665 | 2.22E-04 |
| Il33 | -11.6919 | 1.2797 | 0.073 | 4.80E-05 | 8.99E-07 | 1305.154 | 2.52E-04 |
| Fmnl2 | -10.4992 | 1.4175 | 0.2286 | 2.57E-01 | 9.06E-07 | 3561.3818 | 2.52E-04 |
| Dlg5 | -11.4131 | 0.9988 | 0.2912 | 4.50E-05 | 1.15E-06 | 1470.6879 | 3.06E-04 |
| Pcp4l1 | -12.0747 | 1.3889 | 0.1086 | 4.30E-05 | 1.60E-06 | 1123.0321 | 3.91E-04 |
| Atoh8 | -12.1887 | 1.5334 | 0.6037 | 9.18E-02 | 1.61E-06 | 1207.2186 | 3.91E-04 |
| Hivep3 | -11.2598 | 1.1941 | 0.2655 | 1.35E-01 | 3.03E-06 | 1867.9604 | 6.71E-04 |
| Npr2 | -11.1479 | 0.9032 | 1.0461 | 6.97E-02 | 3.28E-06 | 1769.0849 | 7.01E-04 |
| Smo | -11.4111 | 0.904 | 3.2394 | 1.49E-04 | 4.34E-06 | 1430.6875 | 8.82E-04 |
| Tbx20 | -11.5629 | 1.5241 | 0.1173 | 2.17E-01 | 4.42E-06 | 1706.8904 | 8.83E-04 |
| Maf | -11.1393 | 1.1805 | 0.0335 | 5.75E-05 | 4.54E-06 | 1548.4756 | 8.92E-04 |
| Zfp74 | -12.7691 | 1.8977 | 0.473 | 1.67E-01 | 4.73E-06 | 994.8996 | 9.09E-04 |
| Klhl29 | -11.2074 | 0.9195 | 0.1684 | 6.38E-05 | 5.29E-06 | 1626.8505 | 9.91E-04 |
| Pard6g | -13.5426 | 2.93 | 0.0872 | 3.62E-01 | 6.97E-06 | 1138.3367 | 1.23E-03 |
| Ccl21a | -14.9344 | 5.371 | 0.0027 | 1.76E-04 | 8.29E-06 | 500.9525 | 1.37E-03 |
| Pvt1 | -11.1261 | 1.0346 | 0.6061 | 1.30E-01 | 8.42E-06 | 1902.3245 | 1.38E-03 |
| Comp | -12.7877 | 1.9181 | 0.0377 | 5.49E-05 | 1.03E-05 | 844.313 | 1.64E-03 |
| Ifit1 | -12.2564 | 1.6398 | 0.0762 | 1.91E-01 | 1.84E-05 | 1120.375 | 2.56E-03 |
| Dusp2 | -11.9079 | 1.3348 | 0.0431 | 8.29E-05 | 1.89E-05 | 1098.5902 | 2.60E-03 |
| Parp14 | -10.46 | 0.9668 | 1.6224 | 1.84E-01 | 2.14E-05 | 2913.8645 | 2.87E-03 |
| Kdm4a | -12.0858 | 1.2965 | 2813854.359 | 1.43E-01 | 2.43E-05 | 1076.4189 | 3.20E-03 |
| Nlrc5 | -11.0623 | 0.7172 | 1.1636 | 4.01E-05 | 2.76E-05 | 1691.9019 | 3.52E-03 |
| Duox2 | -13.7284 | 2.5604 | 0.0453 | 8.10E-05 | 3.01E-05 | 667.2265 | 3.79E-03 |
| Nectin2 | -11.3727 | 1.2736 | 0.3227 | 2.27E-01 | 3.17E-05 | 1918.9692 | 3.93E-03 |
| Lcn2 | -12.035 | 1.3827 | 0.0315 | 4.63E-05 | 3.71E-05 | 995.7003 | 4.41E-03 |
| Fmo1 | -9.3223 | 0.7651 | 1.6547 | 1.25E-01 | 3.84E-05 | 5285.7991 | 4.42E-03 |
| Cp | -9.6521 | 1.494 | 0.4069 | 4.21E-01 | 4.04E-05 | 5712.9131 | 4.52E-03 |
| Etfbkmt | -12.2125 | 1.2458 | 0.1318 | 9.76E-05 | 4.21E-05 | 928.4544 | 4.60E-03 |
| Meox1 | -13.4521 | 2.2002 | 0.0765 | 6.94E-02 | 4.24E-05 | 676.7075 | 4.61E-03 |
| Rtp4 | -11.4818 | 0.8728 | 0.3514 | 8.06E-05 | 4.45E-05 | 1329.3381 | 4.72E-03 |
| Gas7 | -13.1641 | 2.1675 | 0.0718 | 1.89E-01 | 4.58E-05 | 816.2447 | 4.79E-03 |
| 9930111J21Rik2 | -11.6267 | 0.8891 | 2.767 | 7.16E-05 | 5.02E-05 | 1232.5102 | 5.08E-03 |

**Endothelial Cell: gene expression, negative correlation (β_1_) with age**

| gene | Beta0 | Beta1 | Disp | Sigma | P.Beta | AIC | fdrP |
| --- | --- | --- | --- | --- | --- | --- | --- |
| Meg3 | -8.9562 | -1.5149 | 0.1679 | 7.61E-05 | 4.70E-32 | 2790.465 | 8.21E-28 |
| Dse | -10.1896 | -1.7585 | 0.2135 | 4.44E-05 | 6.22E-18 | 1115.608 | 3.28E-14 |
| Gap43 | -10.485 | -2.5123 | 1.6542 | 3.49E-05 | 7.50E-18 | 754.1488 | 3.28E-14 |
| Mdga1 | -10.6608 | -1.9318 | 0.3932 | 3.77E-05 | 9.47E-14 | 774.9051 | 1.84E-10 |
| Spag1 | -9.7824 | -1.6218 | 0.0334 | 5.91E-05 | 7.12E-12 | 1220.209 | 1.00E-08 |
| Gm16104 | -10.5292 | -1.3382 | 0.6369 | 6.72E-05 | 1.54E-11 | 1016.566 | 1.93E-08 |
| Srrm3 | -11.0673 | -2.1301 | 0.1121 | 5.07E-05 | 2.64E-09 | 532.0859 | 2.31E-06 |
| Gm43684 | -10.1896 | -4.0471 | 0.005 | 7.60E-05 | 7.64E-09 | 372.3594 | 5.34E-06 |
| Gm31615 | -11.2316 | -2.7864 | 0.2217 | 4.55E-05 | 8.79E-09 | 428.2712 | 5.92E-06 |
| Igf2bp3 | -10.9452 | -1.3142 | 0.3791 | 8.03E-05 | 6.80E-08 | 749.2149 | 3.13E-05 |
| Rian | -10.4354 | -1.0951 | 0.1169 | 4.98E-05 | 9.27E-08 | 1134.834 | 4.05E-05 |
| Klb | -11.2667 | -3.2667 | 0.1104 | 4.30E-05 | 9.83E-08 | 371.3677 | 4.19E-05 |
| Ticam1 | -10.4329 | -1.2247 | 0.0648 | 6.52E-05 | 1.04E-07 | 1025.685 | 4.32E-05 |
| CT030142.7 | -11.4134 | -2.136 | 0.1493 | 4.73E-05 | 2.14E-07 | 414.804 | 8.13E-05 |
| Gm15457 | -10.4653 | -3.593 | 0.0045 | 6.46E-05 | 2.64E-07 | 329.2919 | 9.42E-05 |
| Stra6 | -10.8711 | -1.1789 | 0.2289 | 4.04E-05 | 3.64E-07 | 803.5254 | 1.18E-04 |
| Nudt17 | -11.1315 | -1.2457 | 5.1553 | 1.12E-04 | 7.23E-07 | 649.5678 | 2.14E-04 |
| Mbd4 | -9.789 | -0.9233 | 0.0582 | 4.71E-04 | 8.73E-07 | 1680.194 | 2.50E-04 |
| Vamp9 | -11.5449 | -1.7863 | 0.4355 | 3.97E-06 | 1.96E-06 | 404.2321 | 4.70E-04 |
| Eno2 | -11.445 | -3.5007 | 0.0893 | 4.67E-05 | 2.49E-06 | 320.0683 | 5.73E-04 |
| Ank3 | -9.5097 | -1.5983 | 0.0184 | 2.09E-01 | 3.90E-06 | 1203.792 | 8.22E-04 |
| Kif11 | -11.0057 | -1.6489 | 0.0228 | 6.95E-05 | 4.26E-06 | 545.6294 | 8.76E-04 |
| Ret | -11.0593 | -1.1056 | 0.2873 | 3.61E-05 | 6.05E-06 | 719.1942 | 1.10E-03 |
| Fam189a1 | -11.5805 | -1.9438 | 0.0632 | 4.33E-05 | 7.78E-06 | 372.0498 | 1.33E-03 |
| Gm13708 | -11.7549 | -1.9349 | 2545151.63 | 2.87E-08 | 8.24E-06 | NA | 1.37E-03 |
| Frmd8os | -11.5183 | -1.6904 | 0.0866 | 4.23E-05 | 9.21E-06 | 411.6224 | 1.48E-03 |
| Acpp | -10.9401 | -0.9424 | 0.5096 | 3.05E-05 | 1.25E-05 | 808.7255 | 1.91E-03 |
| Mirg | -11.6553 | -2.2018 | 0.03 | 5.07E-05 | 1.60E-05 | 307.2475 | 2.36E-03 |
| Gm17655 | -10.3516 | -1.3224 | 0.0169 | 1.11E-04 | 2.65E-05 | 826.1289 | 3.44E-03 |
| Zfp935 | -10.7324 | -3.1933 | 0.0991 | 6.32E-01 | 2.72E-05 | 804.5036 | 3.50E-03 |
| Gm13427 | -11.765 | -1.7784 | 0.1355 | 3.88E-05 | 3.23E-05 | 331.156 | 3.98E-03 |
| S100a4 | -9.5423 | -1.8841 | 0.399 | 5.12E-01 | 3.25E-05 | 1796.671 | 3.98E-03 |
| Car7 | -10.88 | -1.6114 | 0.0141 | 6.18E-05 | 3.28E-05 | 530.819 | 3.98E-03 |
| Gm16845 | -10.8646 | -0.8585 | 0.3723 | 8.18E-05 | 3.47E-05 | 904.1943 | 4.18E-03 |
| Ngef | -11.8015 | -1.637 | 3338814.61 | 5.36E-05 | 3.97E-05 | 360.6693 | 4.51E-03 |
| Ip6k3 | -11.1053 | -0.9672 | 0.5368 | 3.70E-05 | 4.06E-05 | 740.3172 | 4.52E-03 |
| Borcs5 | -10.8283 | -0.7906 | 0.8318 | 3.27E-05 | 4.76E-05 | 944.5517 | 4.90E-03 |
| Gpr19 | -10.5417 | -1.0473 | 0.0373 | 1.01E-04 | 4.92E-05 | 927.5052 | 5.00E-03 |
| Gm18227 | -11.2408 | -4.3464 | 0.0118 | 5.61E-05 | 5.07E-05 | 278.1743 | 5.09E-03 |
| Gm45030 | -11.8043 | -2.6841 | 0.0263 | 5.07E-05 | 5.37E-05 | 255.3514 | 5.37E-03 |
| F630040K05Rik | -11.826 | -1.7347 | 0.1538 | 6.03E-05 | 5.96E-05 | 328.6836 | 5.84E-03 |
| Carf | -10.2848 | -1.0342 | 0.0285 | 6.07E-05 | 6.70E-05 | 1033.324 | 6.34E-03 |
| Ninj2 | -11.3841 | -1.3715 | 0.0495 | 9.25E-05 | 7.83E-05 | 494.5388 | 7.04E-03 |
| 4933439C10Rik | -10.9096 | -0.955 | 0.0917 | 4.66E-05 | 7.84E-05 | 817.6342 | 7.04E-03 |
| Efcab12 | -11.6709 | -1.5618 | 0.0649 | 3.79E-05 | 7.84E-05 | 367.9292 | 7.04E-03 |
| Igsf23 | -11.7105 | -1.3603 | 2548673.02 | 9.99E-23 | 8.85E-05 | 404.8744 | 7.70E-03 |
| Gm16157 | -11.9345 | -2.9914 | 0.0546 | 5.24E-05 | 9.25E-05 | 232.8885 | 7.93E-03 |
| Fkbp6 | -12.0942 | -2.4349 | 0.2227 | 1.78E-12 | 1.30E-04 | 221.0568 | 1.00E-02 |
| Gm35215 | -11.9926 | -2.9312 | 0.0486 | 5.10E-05 | 1.38E-04 | 220.3579 | 1.06E-02 |
| 4930506C21Rik | -12.0782 | -2.1644 | 0.1406 | 3.59E-05 | 1.45E-04 | 237.2944 | 1.10E-02 |

**Supplemental Table S5:** NICHES table. Common gene signaling pairs followed by significant gene pairs associated with age-related adventitial remodeling in specific cell-type sender-receiving analyses.


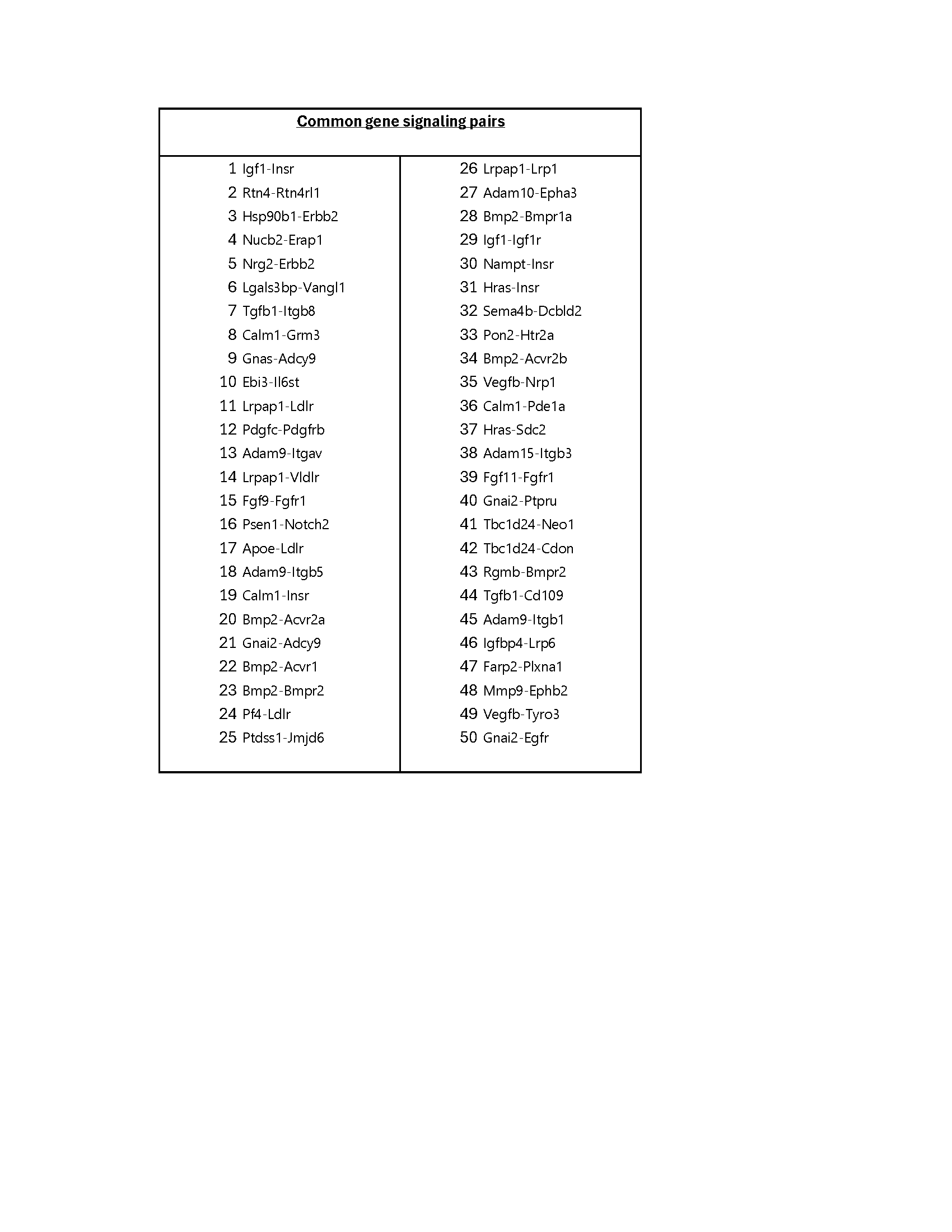


| **Signaling cell types** | **Signaling gene pair** | **p_val** | **avg_log2FC** | **pct.1** | **pct.2** | **p_val_adj** |
| --- | --- | --- | --- | --- | --- | --- |
| MacPeriVasc-Fibroblast | Calm1-Grm3 | 2.18E-59 | 4.50668425 | 0.716 | 0.048 | 2.47E-56 |
| MacPeriVasc-Fibroblast | Lgals3bp-Vangl1 | 4.42E-58 | 3.69389279 | 0.749 | 0.143 | 5.02E-55 |
| MacPeriVasc-Fibroblast | Il18-Il1rl2 | 4.81E-52 | 4.19162667 | 0.652 | 0.028 | 5.46E-49 |
| MacPeriVasc-Fibroblast | Nucb2-Erap1 | 8.47E-50 | 2.91340507 | 0.72 | 0.159 | 9.62E-47 |
| MacPeriVasc-Fibroblast | Igf1-Insr | 5.21E-49 | 3.6012284 | 0.655 | 0.048 | 5.91E-46 |
| MacPeriVasc-Fibroblast | Rtn4-Rtn4rl1 | 4.18E-48 | 3.54725637 | 0.643 | 0.071 | 4.75E-45 |
| MacPeriVasc-Fibroblast | Hsp90b1-Erbb2 | 2.17E-43 | 7.16589959 | 0.54 | 0.008 | 2.46E-40 |
| MacPeriVasc-Fibroblast | Vegfb-Nrp1 | 2.28E-42 | 3.10889271 | 0.637 | 0.103 | 2.59E-39 |
| MacPeriVasc-Fibroblast | Tgfb1-Itgb8 | 6.32E-42 | 3.69395814 | 0.585 | 0.04 | 7.18E-39 |
| MacPeriVasc-Fibroblast | Lrpap1-Vldlr | 1.68E-41 | 1.68279563 | 0.813 | 0.341 | 1.91E-38 |
| MacPeriVasc-Fibroblast | Ptdss1-Jmjd6 | 1.77E-41 | 3.43108261 | 0.614 | 0.091 | 2.01E-38 |
| MacPeriVasc-Fibroblast | Gnas-Adcy9 | 1.79E-41 | 2.68619074 | 0.673 | 0.103 | 2.03E-38 |
| MacPeriVasc-Fibroblast | Nrg2-Erbb2 | 4.19E-41 | 6.92423735 | 0.519 | 0.008 | 4.75E-38 |
| MacPeriVasc-Fibroblast | Pdgfc-Pdgfrb | 6.80E-41 | 2.05059091 | 0.738 | 0.278 | 7.72E-38 |
| MacPeriVasc-Fibroblast | Lrpap1-Lrp1 | 6.93E-41 | 1.37169841 | 0.871 | 0.417 | 7.87E-38 |
| MacPeriVasc-Fibroblast | Bmp2-Acvr2b | 3.52E-40 | 5.04032424 | 0.535 | 0.032 | 4.00E-37 |
| MacPeriVasc-Fibroblast | Igf1-Igf1r | 1.54E-39 | 1.59673705 | 0.801 | 0.345 | 1.74E-36 |
| MacPeriVasc-Fibroblast | Fgf9-Fgfr1 | 3.38E-38 | 2.62422958 | 0.639 | 0.147 | 3.83E-35 |
| MacPeriVasc-Fibroblast | Ebi3-Il6st | 2.43E-37 | 1.46867229 | 0.853 | 0.437 | 2.76E-34 |
| MacPeriVasc-Fibroblast | Adam9-Itgb5 | 4.90E-37 | 1.24213965 | 0.851 | 0.512 | 5.56E-34 |
| MacPeriVasc-SMC | Pon2-Htr2a | 1.11E-55 | 12.3695293 | 0.835 | 0 | 1.26E-52 |
| MacPeriVasc-SMC | Psen1-Notch3 | 9.87E-51 | 1.87000412 | 0.946 | 0.64 | 1.12E-47 |
| MacPeriVasc-SMC | Rtn4-Rtn4rl1 | 1.82E-50 | 3.98633107 | 0.838 | 0.093 | 2.07E-47 |
| MacPeriVasc-SMC | Igf1-Insr | 5.96E-48 | 2.01221948 | 0.937 | 0.379 | 6.77E-45 |
| MacPeriVasc-SMC | Hsp90b1-Erbb2 | 1.17E-47 | 3.72958346 | 0.793 | 0.019 | 1.33E-44 |
| MacPeriVasc-SMC | Nrg2-Erbb2 | 3.86E-47 | 6.36496756 | 0.763 | 0.006 | 4.38E-44 |
| MacPeriVasc-SMC | Gnai2-Ednra | 6.23E-46 | 2.03910658 | 0.931 | 0.236 | 7.07E-43 |
| MacPeriVasc-SMC | Gas6-Tyro3 | 1.24E-45 | 3.91559481 | 0.802 | 0.068 | 1.41E-42 |
| MacPeriVasc-SMC | Vegfb-Tyro3 | 1.34E-45 | 4.87777246 | 0.769 | 0.043 | 1.52E-42 |
| MacPeriVasc-SMC | Adam9-Itgav | 2.53E-45 | 2.41117178 | 0.877 | 0.273 | 2.87E-42 |
| MacPeriVasc-SMC | Psen1-Notch2 | 1.11E-44 | 2.42924246 | 0.862 | 0.304 | 1.25E-41 |
| MacPeriVasc-SMC | Pros1-Tyro3 | 1.64E-44 | 3.21236493 | 0.829 | 0.143 | 1.86E-41 |
| MacPeriVasc-SMC | Tgfb1-Itgb8 | 1.04E-43 | 2.38767586 | 0.865 | 0.13 | 1.19E-40 |
| MacPeriVasc-SMC | Nucb2-Erap1 | 1.49E-43 | 2.89834954 | 0.826 | 0.099 | 1.69E-40 |
| MacPeriVasc-SMC | Adam9-Itga3 | 2.63E-43 | 1.74367441 | 0.895 | 0.565 | 2.99E-40 |
| MacPeriVasc-SMC | Rtn4-Cntnap1 | 6.80E-43 | 4.39299447 | 0.757 | 0.056 | 7.72E-40 |
| MacPeriVasc-SMC | Lrpap1-Ldlr | 7.51E-42 | 4.72438182 | 0.73 | 0.037 | 8.52E-39 |
| MacPeriVasc-SMC | Lgals3bp-Vangl1 | 1.12E-41 | 11.2006616 | 0.691 | 0 | 1.27E-38 |
| MacPeriVasc-SMC | Ebi3-Il6st | 2.79E-40 | 2.67500225 | 0.826 | 0.087 | 3.16E-37 |
| MacPeriVasc-SMC | Gnas-Adcy1 | 5.63E-38 | 2.95380927 | 0.79 | 0.106 | 6.39E-35 |
| Myeloid-Fibroblast | Calm1-Grm3 | 1.12E-27 | 4.11495659 | 0.687 | 0.062 | 1.27E-24 |
| Myeloid-Fibroblast | Il18-Il1rl2 | 3.18E-22 | 2.62914435 | 0.662 | 0.069 | 3.61E-19 |
| Myeloid-Fibroblast | Sema4d-Erbb2 | 3.29E-21 | 12.2715709 | 0.503 | 0 | 3.74E-18 |
| Myeloid-Fibroblast | Rtn4-Rtn4rl1 | 5.83E-21 | 3.06125389 | 0.59 | 0.077 | 6.62E-18 |
| Myeloid-Fibroblast | Lrpap1-Lrp1 | 1.50E-20 | 2.21041281 | 0.662 | 0.115 | 1.70E-17 |
| Myeloid-Fibroblast | Lrpap1-Vldlr | 1.83E-20 | 2.68818771 | 0.631 | 0.1 | 2.07E-17 |
| Myeloid-Fibroblast | Tgfb1-Itgb6 | 9.12E-20 | -2.4542809 | 0.154 | 0.631 | 1.03E-16 |
| Myeloid-Fibroblast | Tgfb1-Itgb8 | 2.33E-19 | 3.06042542 | 0.564 | 0.046 | 2.65E-16 |
| Myeloid-Fibroblast | Hsp90b1-Erbb2 | 3.97E-19 | 11.5887875 | 0.462 | 0 | 4.50E-16 |
| Myeloid-Fibroblast | Lgals3bp-Vangl1 | 1.20E-18 | 4.95842559 | 0.492 | 0.031 | 1.36E-15 |
| Myeloid-Fibroblast | Hras-Insr | 2.34E-18 | 3.34291156 | 0.497 | 0.023 | 2.66E-15 |
| Myeloid-Fibroblast | Fn1-Itgb6 | 2.73E-18 | -3.4745921 | 0.077 | 0.485 | 3.10E-15 |
| Myeloid-Fibroblast | Ptdss1-Jmjd6 | 4.55E-17 | 3.75672188 | 0.508 | 0.077 | 5.16E-14 |
| Myeloid-Fibroblast | Gnas-Adcy9 | 6.51E-17 | 1.86430333 | 0.692 | 0.185 | 7.39E-14 |
| Myeloid-Fibroblast | Calm1-Insr | 2.74E-16 | 1.24128035 | 0.667 | 0.115 | 3.11E-13 |
| Myeloid-Fibroblast | Nampt-Insr | 7.34E-16 | 1.21592471 | 0.626 | 0.108 | 8.33E-13 |
| Myeloid-Fibroblast | Calm2-Insr | 1.36E-15 | 0.83951521 | 0.605 | 0.092 | 1.54E-12 |
| Myeloid-Fibroblast | Hras-Sdc2 | 1.28E-14 | 1.91065434 | 0.59 | 0.154 | 1.46E-11 |
| Myeloid-Fibroblast | Mfng-Notch2 | 1.35E-14 | 2.02276097 | 0.508 | 0.077 | 1.53E-11 |
| Myeloid-Fibroblast | S100a8-Tlr4 | 3.93E-14 | 4.39464445 | 0.405 | 0.031 | 4.46E-11 |
| Myeloid-SMC | Gnai2-Ednra | 2.69E-22 | 2.4809227 | 0.902 | 0.239 | 3.06E-19 |
| Myeloid-SMC | Pon2-Htr2a | 4.39E-22 | 11.1998938 | 0.75 | 0 | 4.99E-19 |
| Myeloid-SMC | Rtn4-Rtn4rl1 | 8.69E-21 | 4.07306333 | 0.774 | 0.099 | 9.86E-18 |
| Myeloid-SMC | Pros1-Tyro3 | 1.25E-20 | 3.68875145 | 0.78 | 0.056 | 1.42E-17 |
| Myeloid-SMC | Psen1-Notch3 | 1.17E-19 | 1.30242219 | 0.939 | 0.746 | 1.33E-16 |
| Myeloid-SMC | Sema4d-Erbb2 | 1.39E-19 | 3.6331958 | 0.744 | 0.028 | 1.58E-16 |
| Myeloid-SMC | Lrpap1-Sort1 | 2.43E-19 | 3.77776103 | 0.756 | 0.085 | 2.76E-16 |
| Myeloid-SMC | Tgfb1-Itgb8 | 6.48E-19 | 2.61098911 | 0.823 | 0.169 | 7.35E-16 |
| Myeloid-SMC | Rtn4-Cntnap1 | 7.72E-19 | 4.72944892 | 0.713 | 0.042 | 8.76E-16 |
| Myeloid-SMC | Hsp90b1-Erbb2 | 2.58E-18 | 4.82731371 | 0.689 | 0.014 | 2.92E-15 |
| Myeloid-SMC | Adam9-Itga3 | 2.72E-17 | 1.63797058 | 0.866 | 0.38 | 3.08E-14 |
| Myeloid-SMC | Adam9-Itgav | 5.55E-17 | 1.96653111 | 0.841 | 0.169 | 6.30E-14 |
| Myeloid-SMC | Lrpap1-Vldlr | 9.24E-17 | 2.89551516 | 0.756 | 0.113 | 1.05E-13 |
| Myeloid-SMC | Lrpap1-Sorl1 | 2.62E-16 | 2.74905962 | 0.756 | 0.127 | 2.97E-13 |
| Myeloid-SMC | Psen1-Notch2 | 2.83E-16 | 1.70210574 | 0.848 | 0.394 | 3.21E-13 |
| Myeloid-SMC | Lrpap1-Lrp1 | 4.31E-16 | 2.7650378 | 0.744 | 0.099 | 4.89E-13 |
| Myeloid-SMC | Gnas-Adcy1 | 5.47E-16 | 2.59588551 | 0.774 | 0.169 | 6.21E-13 |
| Myeloid-SMC | Ebi3-Il6st | 6.65E-16 | 1.97339823 | 0.732 | 0.07 | 7.55E-13 |
| Myeloid-SMC | Calm1-Grm3 | 7.04E-16 | 2.22203503 | 0.823 | 0.169 | 7.99E-13 |
| Myeloid-SMC | Lrpap1-Ldlr | 8.78E-16 | 5.21552699 | 0.616 | 0.014 | 9.97E-13 |

**References**

1. Elassal A, Steppan J, Charania S, Santhanam L, Singh I, Heerdt PM. Pressure-based estimation of right ventricular ejection fraction: Validation as a clinically relevant target for drug development in a rodent model of pulmonary hypertension. Journal of Pharmacological and Toxicological Methods. 2021;112:107102.

2. Gardi C, Martorana P, Calzoni P, Cavarra E, Marcolongo P, de Santi M, van Even P, Lungarella G. Cardiac collagen changes during the development of right ventricular hypertrophy in tight-skin mice with emphysema. Experimental and molecular pathology. 1994;60(2):100-7.

3. Miles C, Westaby J, Ster IC, Asimaki A, Boardman P, Joshi A, Papadakis M, Sharma S, Behr ER, Sheppard MN. Morphometric characterization of collagen and fat in normal ventricular myocardium. Cardiovascular Pathology. 2020;48:107224.

4. Zhou P, Pu WT. Recounting Cardiac Cellular Composition. Circ Res. 2016;118(3):368-70.

5. EGHBALI M, EGHBALI M, ROBINSON TF, SEIFTER S, BLUMENFELD OO. Collagen accumulation in heart ventricles as a function of growth and aging. Cardiovascular research. 1989;23(8):723-9.

6. Ridderbos F-JS, Wolff D, Timmer A, van Melle JP, Ebels T, Dickinson MG, Timens W, Berger RM. Adverse pulmonary vascular remodeling in the Fontan circulation. The Journal of Heart and Lung Transplantation. 2015;34(3):404-13.

7. Schulte H, Mühlfeld C, Brandenberger C. Age-Related Structural and Functional Changes in the Mouse Lung. Frontiers in Physiology. 2019;10.

8. Dagnachew YM, Lim HY, Wupeng L, Lim SY, Lim SJN, Thiam CH, Tan SW, Eng JLJ, Mei D, Hazwany Mohammad Azhar S. Collagen deposition in lung parenchyma driven by depletion of interstitial Lyve-1+ macrophages prevents cigarette smoke-induced emphysema and loss of airway function. Frontiers in Immunology. 2025;15:1493395.

9. Andrikakou P, Vickraman K, Arora H. On the behaviour of lung tissue under tension and compression. Sci Rep 6: 36642. 2016.

10. Mercer RR, Crapo JD. Spatial distribution of collagen and elastin fibers in the lungs. Journal of Applied Physiology. 1990;69(2):756-65.

11. Chrzanowski P, Keller S, Cerreta J, Mandl I, Turino G. Elastin content of normal and emphysematous lung parenchyma. The American journal of medicine. 1980;69(3):351-9.

12. Pierce JA, Ebert RV. Fibrous network of the lung and its change with age. Thorax. 1965;20(5):469.

13. Vindin HJ, Oliver BG, Weiss AS. Elastin in healthy and diseased lung. Current opinion in biotechnology. 2022;74:15-20.

14. Huang K, Rabold R, Schofield B, Mitzner W, Tankersley CG. Age-dependent changes of airway and lung parenchyma in C57BL/6J mice. Journal of Applied Physiology. 2007;102(1):200-6.

15. Wang B, Wang L, Gasek NS, Zhou Y, Kim T, Guo C, Jellison ER, Haynes L, Yadav S, Tchkonia T, Kuchel GA, Kirkland JL, Xu M. An inducible p21-Cre mouse model to monitor and manipulate p21-highly-expressing senescent cells in vivo. Nat Aging. 2021;1(10):962-73. Epub 20211007.

16. Wang Z, Rong X, Luo B, Qin S, Lu L, Zhang X, Sun Y, Hu Q, Zhang C. A Natural Model of Mouse Cardiac Myocyte Senescence. J Cardiovasc Transl Res. 2016;9(5-6):456-8. Epub 20160915.

17. Yao H, Wallace J, Peterson AL, Scaffa A, Rizal S, Hegarty K, Maeda H, Chang JL, Oulhen N, Kreiling JA, Huntington KE, De Paepe ME, Barbosa G, Dennery PA. Timing and cell specificity of senescence drives postnatal lung development and injury. Nat Commun. 2023;14(1):273. Epub 20230117.

18. Meca-Laguna G, Qiu M, Hou Y, Barkovskaya A, Shankar A, Dixit B, Rae MJ, Boominathan A, Sharma A. Cell-Surface LAMP1 is a Senescence Marker in Aging and Idiopathic Pulmonary Fibrosis. Aging Cell. 2025;24(9):e70141. Epub 20250622. doi: 10.1111/acel.70141. PubMed PMID: 40545776; PMCID: PMC12419843.

19. Rashid K, Sundar IK, Gerloff J, Li D, Rahman I. Lung cellular senescence is independent of aging in a mouse model of COPD/emphysema. Scientific Reports. 2018;8(1):9023.

11. Eghbali M, Eghbali M, Robinson TF, Seifter S, Blumenfeld OO. Collagen accumulation in heart ventricles as a function of growth and aging. Cardiovascular research. 1989;23(8):723-9.
